# The dynamin-related protein Vps1 and the peroxisomal membrane protein Pex27 function together during peroxisome fission

**DOI:** 10.1242/jcs.246348

**Published:** 2023-03-24

**Authors:** Lakhan Ekal, Abdulaziz M. S. Alqahtani, Ewald H. Hettema

**Affiliations:** ^1^ School of Biosciences, University of Sheffield, Sheffield S10 2TN, UK; ^2^ Department of Biology, Faculty of Science, University of Bisha, P.O. Box 551, Bisha 61922, Saudi Arabia

**Keywords:** Dynamin-related protein, Peroxisome, Autophagy, Drp1

## Abstract

Dynamin-related proteins (Drps) mediate a variety of membrane remodelling processes. The *Saccharomyces cerevisiae* Drp, Vps1, is required for endocytosis, endosomal sorting, vacuole fusion, and peroxisome fission and breakdown. How Drps, and in particular Vps1, can function at so many different subcellular locations is of interest to our understanding of cellular organisation. We found that the peroxisomal membrane protein Pex27 is specifically required for Vps1-dependent peroxisome fission in proliferating cells but is not required for Dnm1-dependent peroxisome fission. Pex27 accumulates in constricted regions of peroxisomes and affects peroxisome geometry upon overexpression. Moreover, Pex27 physically interacts with Vps1 *in vivo* and is required for the accumulation of a GTPase-defective Vps1 mutant (K42A) on peroxisomes. During nitrogen starvation, a condition that halts cell division and induces peroxisome breakdown, Vps1 associates with the pexophagophore. Pex27 is neither required for Vps1 recruitment to the pexophagophore nor for pexophagy. Our study identifies Pex27 as a Vps1-specific partner for the maintenance of peroxisome number in proliferating yeast cells.

## Teaser

**Highlighted Article:** Yeast peroxisome fission is mediated by the dynamin-related proteins Vps1 and Dnm1. Pex27 accumulates at peroxisomal membrane constrictions and is specifically required for Vps1 accumulation and activity on peroxisomes.

## INTRODUCTION

Dynamin-related proteins (Drps) comprise a group of self-assembling GTPases that mediate intracellular membrane fission and fusion events. Their activities affect processes such as endocytosis, endosomal protein sorting, and organelle fission and fusion (Ferguson and De Camilli, 2012; Praefcke and McMahon, 2004). Drps contain conserved functional domains including a large GTPase domain. In addition, the founding member of this protein family, dynamin, contains a pleckstrin homology domain (PHD) and a proline-rich domain (PRD) that are required for lipid binding and for interaction with other proteins, respectively (Jimah and Hinshaw, 2019).

The best-studied function of dynamin is in clathrin-mediated endocytosis, where it induces scission of endocytic vesicles from the plasma membrane. *In vitro* studies show that dynamin assembles onto tubulated membranes to form helical polymers that constrict upon GTP binding and further constrict upon GTP hydrolysis to induce fission (reviewed in Antonny et al., 2016). A variety of proteins interact with dynamin at sites of clathrin-mediated endocytosis. These proteins act as adaptors to specifically recruit dynamin and/or regulate its activity. Among these proteins, the bar domain-containing proteins amphiphysin and endophilin generate membrane curvature at the vesicle neck, which allows dynamin polymers to assemble (Ross et al., 2011; Roux et al., 2010; Takei et al., 1999).

Drp1 (Dnm1 in *Saccharomyces cerevisiae*) is required for fission of intracellular organelles such as mitochondria and peroxisomes (reviewed in Jimah and Hinshaw, 2019). In *S. cerevisiae*, a second Drp, Vps1, is involved in peroxisome fission (Hoepfner et al., 2001). Vps1 is also involved in endocytosis (Smaczynska-de Rooji et al., 2010), multiple endosomal trafficking events (Lukehart et al., 2013; Nothwehr et al., 1995; Wilsbach and Payne, 1993), peroxisome breakdown (Mao et al., 2014) and vacuole fusion (Peters et al., 2004). Although related to dynamin, it lacks a PHD and PRD. Instead, it contains a region that varies among the Drps, called insert B (Varlakhanova et al., 2018). Insert B of Vps1 has been proposed to functionally resemble the PHD of dynamin (Smaczynska-de Rooji et al., 2019). As described for dynamin, Vps1 assembles on lipid nanotubes *in vitro* and interacts with membrane curvature-inducing proteins such as amphiphysin (Rvs167 in *S. cerevisiae*) and the bar domain-containing sorting nexin Mvp1 *in vivo* (Chi et al., 2014; Ma et al., 2017; Smaczynska-de Rooji et al., 2012). Mvp1 has been shown to tubulate endosomal membranes and recruit Vps1 to sites of fission (Suzuki et al., 2021). There is a longstanding interest in the identification of factors that contribute to dynamin and Drp-dependent functions in order to fully understand how these proteins execute a wide variety of functions on different target membranes, including the peroxisomal membrane.

Vps1 is the main Drp that mediates peroxisome fission in dividing *S. cerevisiae* cells, with only a minor contribution of Dnm1 (Hoepfner et al., 2001; Kuravi et al., 2006; Motley and Hettema, 2007). Dnm1-dependent peroxisome fission relies on the cofactors Fis1, Mdv1 and Caf4, which recruit and regulate Dnm1 activity. The Dnm1 cofactors are not required for Vps1-dependent peroxisome fission (Motley et al., 2008). When peroxisomes are no longer required, especially under conditions of nitrogen starvation, they are removed by pexophagy (Hutchins et al., 1999). The peroxisomal membrane protein Pex3 recruits the pexophagy receptor Atg36. Atg36 connects peroxisomes via Atg11 and Atg8 with the core autophagy machinery (Motley et al., 2012a,b). Efficient pexophagy relies on fission of peroxisomes into small vesicles to allow incorporation into pexophagosomes. Under these conditions, Atg36 and Atg11 are both required to recruit Vps1 to peroxisomes (Mao et al., 2014). Peroxisome number is not reduced in proliferating cells lacking Atg11 or Atg36 (Motley et al., 2012b). This raises the question of how Vps1 activity on peroxisomal membranes is achieved in proliferating cells, as it seems unlikely that Atg11 and Atg36 are required.

Peroxisomes divide by a multistep process that comprises membrane elongation, constriction and fission. Candidate proteins that might contribute to Vps1-dependent peroxisome fission are the Pex11 family of peroxisomal membrane proteins. This family of proteins is conserved among eukaryotes and has been linked to peroxisome division (Honsho et al., 2016; Schrader et al., 2016), and some family members have been implicated in *de novo* formation of peroxisomes (Chang et al., 2015; Huber et al., 2012). In addition, Pex11 family members have been assigned roles in peroxisomal metabolism and membrane contact site with other organelles (reviewed in Carmichael and Schrader, 2022). Loss of Pex11β function in humans leads to disease (Ebberink et al., 2012). Phylogenetic analysis has revealed a complex evolutionary history of the Pex11 family (Chang et al., 2015; Jansen et al., 2021). Several members of the Pex11 family, including Pex11β, contain an amphipathic helix that is required for membrane remodelling activity *in vitro* and peroxisome fission *in vivo* (Opalinski et al., 2011; Su et al., 2018; Yoshida et al., 2015). Pex11 oligomerisation is also important for membrane remodelling and is considered important for membrane tubulation and assembly of the fission machinery (Bonekamp et al., 2013; Itoyama et al., 2012). Pex11β in mammals and Pex11 in *Hansenula polymorpha* interact with Fis1 and Dnm1, which is thought to couple membrane remodelling and Dnm1 recruitment. In addition, Pex11 physically interacts with Dnm1 directly and stimulates its GTPase activity (Schrader et al., 2022; Williams et al., 2015).

In *S. cerevisiae*, the Pex11 family consists of Pex11, Pex25 and Pex27. Pex11 is required for peroxisome proliferation in response to growth on fatty acids such as oleate as the sole carbon source (Erdmann and Blobel, 1995; Marshall et al., 1995) for fatty acid β-oxidation (Mindthoff et al., 2016; van Roermund et al., 2000), and it mediates contacts between mitochondria and peroxisomes (Esposito et al., 2019; Mattiazzi Usaj et al., 2015). Pex25 is a fungal innovation (Chang et al., 2015). Its paralogue, Pex27, is thought to have subsequently arisen during the whole-genome duplication of an ancestor of *S. cerevisiae* (Byrne and Wolfe, 2005) and is found in a subset of yeasts only. Both proteins affect peroxisome number and shape during peroxisome proliferation but also under non-proliferation-inducing conditions (growth on glucose-containing medium) (Rottensteiner et al., 2003; Smith et al., 2002; Tam et al., 2003; Tower et al., 2011). A population of exponentially growing *pex25Δ* cells display multiple defects, including cells with a low number of enlarged peroxisomes, partial mislocalisation of matrix proteins to the cytosol and segregation defects. These segregation defects would normally induce *de novo* peroxisome formation but this process is strongly delayed in *pex25Δ* cells, thereby resulting in cells lacking peroxisomal structures altogether (Huber et al., 2012; Rottensteiner et al., 2003; Smith et al., 2002; Tam et al., 2003). The molecular role of Pex25 in peroxisome dynamics remains unclear, but Pex25 has been shown to initiate elongation and tubulation of the peroxisomal membrane, which has been proposed to be required for both Vps1-dependent and Dnm1-dependent peroxisome fission (Huber et al., 2012). Pex27 is a low-expressed and poorly characterised member of the Pex11 family of proteins. Pex27 is constitutively expressed, whereas Pex11 and Pex25 are further induced on oleate-containing medium. *PEX27* gene deletion reduces peroxisome number (Rottensteiner et al., 2003; Tam et al., 2003; Tower et al., 2011) and Pex27 overexpression has been reported to antagonise Pex25 function (Huber et al., 2012). Pex34 is a distantly related protein to the Pex11 protein family that regulates peroxisome number in concert with Pex11 family proteins (Jansen et al., 2021; Tower et al., 2011).

Here, we report that Pex27 is specifically required for Vps1-dependent peroxisome fission in dividing cells but not for Dnm1-dependent peroxisome fission. We found that Pex27 physically interacted with Vps1 and that accumulation of the Vps1 GTPase-deficient mutant, Vps1-K42A–GFP, on peroxisomes was dependent on Pex27. In a peroxisome fission-deficient mutant, Pex27–mNeonGreen (mNG) localised to constricted sites on the peroxisomal membrane. Overexpression of Pex27 induced an increase in peroxisome number in the presence of Vps1, but in *vps1Δ/dnm1Δ* cells, Pex27 overexpression induced narrow tubules that connected bulbous parts of the peroxisomal structures, resulting in dumbbell-shaped peroxisomes. It was on these tubular connections that Pex27–mNG accumulated. Our data support a model wherein Pex27 recruits Vps1 or facilitates assembly of Vps1 oligomers to constricted sites on the peroxisomal membrane. In addition, we found that Pex27 was not required for pexophagy and recruitment of Vps1 to peroxisomes under pexophagy conditions. This qualifies Pex27 as a conditional cofactor of Vps1 on peroxisomes.

## RESULTS

### Pex27 is required for Vps1-dependent peroxisome fission

**Fig. 1.**
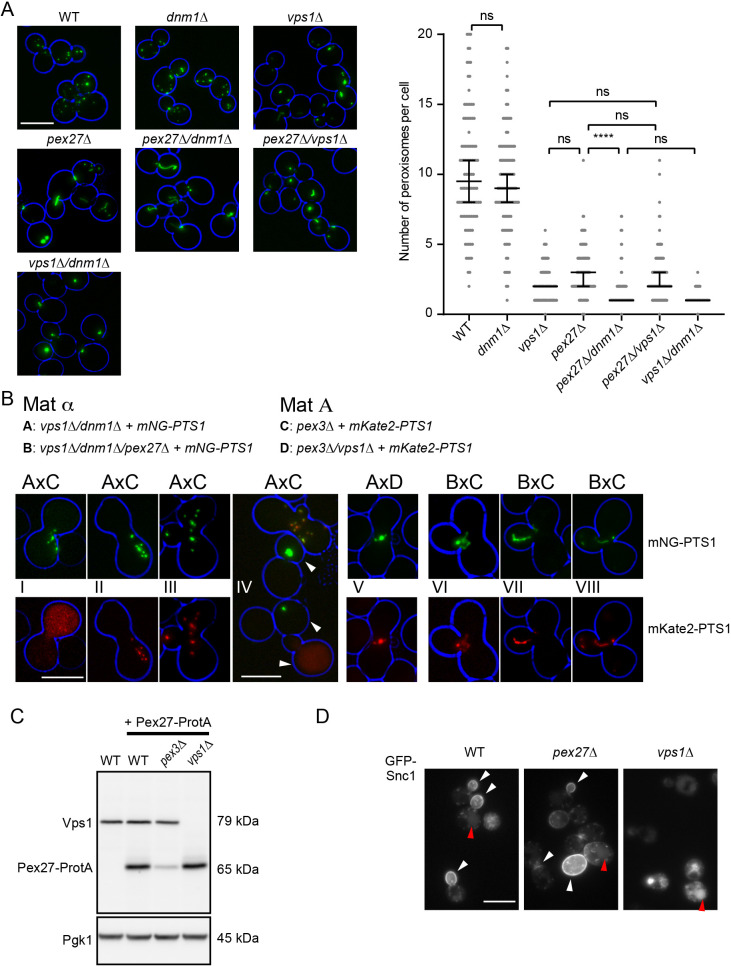
**Pex27 is required for Vps1-dependent peroxisome fission.** (A) Representative epifluorescence microscopy images captured from cells expressing mNeonGreen (mNG)–PTS1 that were grown for extended periods in log phase on 2% glucose-containing medium. Peroxisome numbers were quantified for >100 budding cells for each strain grown. Bars in the graph indicate the median with 95% confidence intervals for mean. Statistical significance analysis was performed using Kruskal–Wallis test. ns, not significant; ****P<0.0001. (B) Peroxisomes in *Matα vps1Δ/dnm1Δ*+mNG–PTS1 and *vps1Δ/dnm1Δ/pex27Δ*+mNG–PTS1 cells were pulse labelled with mNG–PTS1 under control of the inducible *GAL1* promoter (pulse for 3 h with galactose, chase for 2 h with glucose) and mated with MatA *pex3Δ* and *pex3Δ/vps1Δ* cells constitutively expressing mKate2–PTS1 for 2–4 h before imaging. After cell fusion and cytoplasmic mixing, mKate2–PTS1 was imported into the mNG-labelled peroxisomal structures, which, in the presence of Vps1 and Pex27, divided into multiple peroxisomes (panels I–III). Arrowheads in panel IV indicate cells that have not mated. No fission occurred of pre-labelled peroxisomes that lacked Vps1 (panel V) or when the *pex3Δ* mating partner lacked Pex27 (panels VI–VIII). Bright-field images were collected in one plane and processed to highlight the cell circumference in blue. (C) Pex27–ProtA was expressed from a centromeric plasmid under control of its own promoter. This plasmid was transformed into WT, *vps1Δ* and *pex3Δ* cells. Western blot analysis of lysates of the indicated strains using anti-Vps1 and peroxidase anti-peroxidase to detect Vps1 and Pex27–ProtA, respectively, was performed. Pgk1 was used as loading control. (D) Pex27 is not required for GFP–Snc1 recycling through the endosomal system. Representative images were captured from cultures of cells expressing GFP–Snc1 that were grown in log phase on 2% glucose-containing medium. White arrowheads indicate GFP–Snc1 in regions of polarised growth; red arrowheads indicate vacuoles. In A,B,D, images are flattened *z*-stacks. Images are representative of three independent experiments. Scale bars: 5 μm.

In most organisms studied, peroxisome fission relies on a single Drp, Drp1. The fungal Drp, Dnm1, acts in concert with Fis1, Mdv1 and, as shown in *H. polymorpha*, also Pex11. Peroxisome fission in *S. cerevisiae* mainly relies on the Drp, Vps1, and, to a lesser extent, Dnm1. As Vps1-dependent fission is not dependent upon Fis1, Mdv1 and its paralogue Caf4 (Motley et al., 2008; Nagotu et al., 2008), we set out to identify factors specifically required for Vps1-dependent peroxisome fission. We generated double gene-deletion mutants of *PEX11*, *PEX25*, *PEX27* and *PEX34* with either a *VPS1* or a *DNM1* deletion and expressed the monomeric Neon Green (mNG) fluorescent protein appended with a peroxisome-targeting signal type 1 (PTS1), which allows for bright labelling of peroxisomes in living cells, and compared peroxisome number in each of these strains. A factor specifically required for Vps1-dependent fission is expected to: (1) show a strong decrease in peroxisome number when deleted on its own, as is observed in *vps1Δ* cells; (2) have no further decrease in peroxisome number upon *VPS1* deletion; and (3) show a further decrease in peroxisome number upon *DNM1* deletion. We standardised our growth conditions so that we were only analysing cells in the exponential growth phase on glucose-containing medium (see Materials and Methods). An initial screen revealed that only one mutant, *pex27Δ*, fitted our criteria (Fig. S1). A selection of strains was regrown, making sure that overnight cultures did not reach stationary phase before dilution in the morning and had at least 6 h growth in fresh glucose-containing medium. Deletion of *PEX27* resulted in a strong reduction in peroxisome number, which was further significantly reduced in *pex27Δ/dnm1Δ* cells but not in *vps1Δ/pex27Δ* cells (Fig. 1A). The peroxisomes in *pex27Δ/dnm1Δ* cells were mostly elongated, frequently extending from the mother cell into the bud. This phenotype was also observed in *vps1Δ/dnm1Δ* cells (Fig. 1A; Fig. S1). These observations suggest that Pex27 and Vps1 might operate together in the maintenance of peroxisome number.

We used a previously developed mating approach that specifically assays for Vps1-dependent peroxisome fission (Motley and Hettema, 2007) to test for the requirement of Pex27. Haploid *vps1Δ/dnm1Δ* cells pulse labelled with mNG–PTS1 were mated with MatA *pex3Δ* cells expressing mKate2–PTS1. *pex3Δ* cells are devoid of typical peroxisomal membrane structures and many peroxisomal membrane proteins are present at low levels (Hettema et al., 2000; Wroblewska et al., 2017), including Pex27 (Fig. 1C). In this assay, Vps1 from a MatA *pex3Δ* cell diffuses into a Matα *vps1Δ/dnm1Δ* cell and remodels and divides the single pre-labelled peroxisome into multiple smaller ones (Fig. 1B, panels I–IV). Remodelling occurs rapidly upon mating, before the cytosolic mKate2–PTS1 pool becomes evidently punctate (Fig. 1B, panel I). By the time zygotes were formed, all (18/18) zygotes showed multiple dispersed peroxisomes. Dnm1 does not contribute to peroxisome fission under these assay conditions, probably as it is mainly associated with mitochondria and no free pool of Dnm1 is available (Motley and Hettema, 2007; Motley et al., 2008). Indeed, if MatA *pex3Δ* cells additionally lacked *VPS1*, peroxisomes did not divide upon mating and zygotes contained a single peroxisomal structure (15/15 zygotes) (Fig. 1B, panel V) (see also Motley and Hettema, 2007). In Matα *vps1Δ/dnm1Δ* cells lacking *PEX27*, reintroduction of Vps1 upon mating with MatA *pex3Δ* cells did not rescue peroxisome fission before import of mKate2–PTS1 was observed. Even at later stages of mating, when mKate2–PTS1 was clearly imported and zygotes were being formed, we observed elongated peroxisomes in all cells (Fig. 1B, panels V–VII), with 11 out of 15 zygotes containing one or two peroxisomal structures. The remaining four zygotes contained a small number of puncta and elongated peroxisomes (Fig. 1B, panel VIII), suggesting that fission was being restored. The observation that fission was being restored in large zygotes was not completely unexpected as newly synthesised Pex27 would then be routed to the Pex27-deficient pre-existing peroxisome. We conclude that Vps1-dependent peroxisome fission requires Pex27. Vps1 is involved in many membrane remodelling events including protein sorting through the endomembrane system (Lukehart et al., 2013; Nothwehr et al., 1995; Wilsbach and Payne, 1993). The steady-state distribution of GFP–Snc1 is a good marker for recycling through the endosomal system (Lewis et al., 2000). This vesicular SNARE is required for fusion of secretory vesicles with the plasma membrane and is recycled via endosomes to the late Golgi apparatus. As secretion is a polarised process in *S. cerevisiae*, GFP–Snc1 strongly labels the plasma membrane in buds and in the bud neck in cells prior to cytokinesis (Lewis et al., 2000). In *vps1Δ* cells, GFP–Snc1 is not retrieved from endosomes but instead accumulates in the vacuole (Ma et al., 2017). GFP–Snc1 steady-state distribution was unaffected in *pex27Δ* cells (Fig. 1D). These results strongly suggest that Pex27 is a factor specifically required for Vps1-dependent peroxisome multiplication, which is in agreement with its localisation at the peroxisomal membrane (Rottensteiner et al., 2003; Tam et al., 2003).

### Pex27 levels are limiting for Vps1-dependent peroxisome fission

**Fig. 2.**
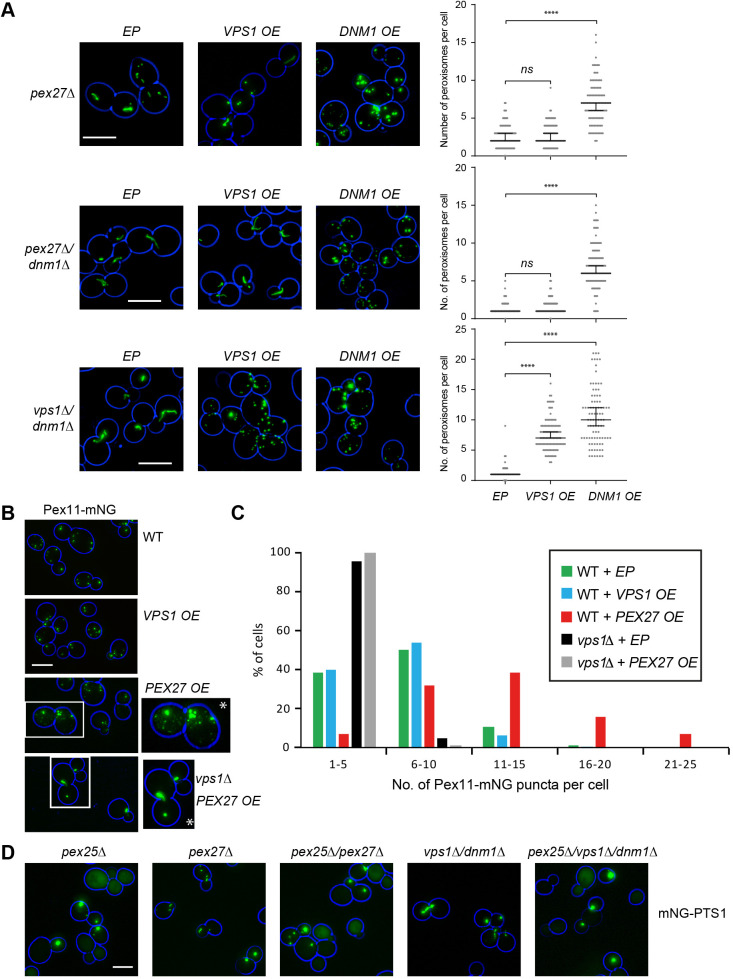
**Pex27 levels are limiting for Vps1-dependent peroxisome fission.** (A) Vps1 overexpression (OE) does not restore peroxisome fission in either *pex27Δ* or *dnm1Δ* /*pex27Δ* cells in contrast to overexpression of Dnm1. Strains expressed mNG–PTS1 from a constitutive promoter. Peroxisome numbers were quantified for >100 (top and middle panels) and >89 (bottom panels) budding cells for each strain grown. Bars in the graph indicate median with 95% confidence intervals for mean. Statistical significance analysis was performed using Kruskal–Wallis test. ns, not significant; ****P<0.0001. (B) Overexpression of Pex27 but not of Vps1 leads to an increase in the number of peroxisomal membrane structures that is dependent on the presence of Vps1. Pex11 was C-terminally tagged with mNG. Boxed cells were further magnified (asterisks) and the level of green fluorescence was enhanced. (C) Quantitation of experiments shown in B in which more than 100 cells were analysed for peroxisome number. (D) Epistatic analysis shows that Pex25 acts upstream of Pex27, Vps1 and Dnm1. Epifluorescence microscopy images captured from cells expressing mNG–PTS1 (A,D) or Pex11–mNG (B,C) that were grown for extended periods on 2% glucose-containing medium are representative of three independent imaging experiments. Images are flattened *z*-stacks. Cell circumference is labelled in blue. Scale bars: 5 μm. EP, empty plasmid control.

Overexpression of Dnm1 but not Vps1 restored peroxisome abundance in *pex27Δ* and *dnm1Δ/pex27Δ* cells (Fig. 2A). This corroborates the model that Pex27 is specifically required for Vps1-dependent peroxisome fission. Although overexpression of Vps1 restored peroxisome number in *vps1Δ/dnm1Δ* cells, it did not induce an increase in peroxisome number in wild-type (WT) cells (Fig. 2B,C; Fig. S2A). This suggests that Vps1 is not limiting for peroxisome fission. Overexpression of Pex27 has previously been reported to interfere with peroxisome functioning by antagonising Pex25 activity (Huber et al., 2012). Indeed, Pex27 overexpression resulted in partial mislocalisation of a peroxisomal matrix marker in some cells, which somewhat resembled *pex25Δ* cells (Fig. S3A). However, when using the peroxisomal membrane proteins Pex11–mNG and Pex13–GFP as markers, we found an increase in peroxisomal membrane structures upon Pex27 overexpression that was dependent upon Vps1 (Fig. 2B,C; Fig. S3B,C). This Pex27 overexpression phenotype is different from *pex25Δ* cells as in *pex25Δ* cells, Pex11–mNG was either localised to the low number of peroxisomes or mislocalised to the tubular network most likely to be mitochondria (Fig. S3D). Pex11 has previously been shown to mistarget to mitochondria in cells that lack peroxisomal membrane structures (Motley et al., 2015). How Pex27 overexpression interferes with matrix protein import is unclear, but as it was unrelated to excessive fission of peroxisomes, this was not further investigated. We conclude that the levels of Pex27 are limiting for Vps1-dependent peroxisome fission.

Epistatic analysis suggested that Pex25 acts upstream of Pex27, Vps1 and Dnm1 as *pex25Δ*/*pex27Δ* and *pex25Δ*/*vps1Δ/dnm1Δ* cells displayed a phenotype similar to *pex25Δ* cells; e.g. cells either lacked peroxisomes or contained a reduced number of spherical peroxisomes, with many cells showing partial mislocalisation of matrix proteins (Fig. 2D; Fig. S1). Overexpression of *VPS1* or *DNM1* did not restore peroxisome number in *pex25Δ* cells (Fig. S2B). Whereas many *pex27Δ*, *vps1Δ* and *vps1Δ/dnm1Δ* mutants displayed tubular peroxisomes, tubular peroxisomes were mostly absent when *PEX25* was deleted in these mutants (Fig. 2D; Fig. S3H,I). This is in agreement with previous studies that proposed a role for Pex25 in peroxisome tubulation (Huber et al., 2012). Although the *pex25Δ* cells that contained peroxisomes localised Pex27–mNG to peroxisomes (Fig. S3F), overexpression of *PEX27* did not induce peroxisome tubulation or multiplication in *pex25Δ* and *vps1Δ/dnm1Δ*/*pex25Δ* cells (Fig. S3D,E,H,I). Therefore, we conclude that Pex27 activity is dependent upon Pex25.

### Vps1 accumulation on peroxisomes requires Pex27

**Fig. 3.**
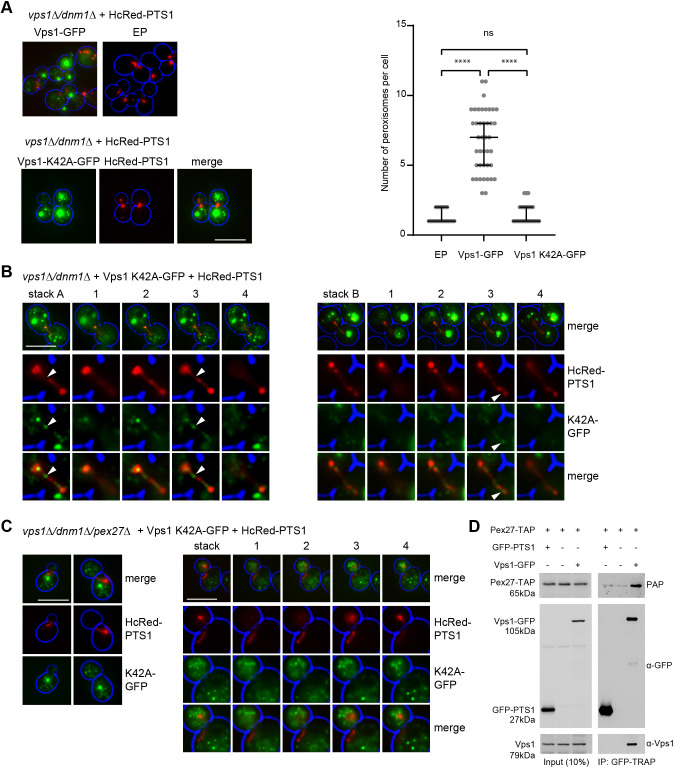
**Vps1 accumulation on peroxisomes requires Pex27.** (A–C) Representative epifluorescence microscopy images of *vps1Δ*/*dnm1Δ* co-expressing HcRed–PTS1 and either Vps1–GFP or Vps1-K42A–GFP. (A) Vps1–GFP but not Vps1-K42A–GFP restores peroxisome fission in *vps1Δ*/*dnm1Δ* cells. Peroxisome numbers were quantified from >28 budding cells for each strain grown. Bars in the graph indicate median with 95% confidence intervals for the mean. Statistical significance analysis was performed using Kruskal–Wallis test. ns, not significant; ****P<0.0001. (B) Vps1-K42A–GFP associates with peroxisomes and was observed along constricted regions of the elongated peroxisome. Stacks ‘A’ and ‘B’ are separate examples of flattened *z*-stacks; the numbers 1–4 indicate individual slices 0.5 µm apart. The bottom three rows are zoomed in. White arrowheads indicate examples of Vps1-K42A–GFP puncta along narrow areas of the peroxisomal tube juxtaposed to more bulbous areas of the peroxisome. (C) Vps1-K42A–GFP does not enrich on peroxisomes in *vps1Δ*/*dnm1Δ*/*pex27Δ* cells. The panels on the right are an example of a flattened *z*-stack; the numbers 1–4 indicate individual slices 0.5 µm apart. The bottom three rows are zoomed in. For A–C, cell circumference is labelled in blue. Scale bars: 5 μm. (D) Co-immunoprecipitation (IP) analysis of Pex27–TAP-expressing strain expressing either Vps1–GFP under control of its own promoter or GFP–PTS1 under control of the *TPI1* promoter from a centromeric plasmid. Vps1–GFP fusion proteins were immunoprecipitated using GFP–nanobody beads (GFP-TRAP, ChromoTek). IP samples and inputs were analysed by western blotting using antibodies against Vps1 and GFP to detect GFP fusion proteins. Pex27–TAP was detected using the peroxidase-anti-peroxidase (PAP) antibody. Pex27–TAP interacts with Vps1–GFP. Vps1–GFP also co-precipitates endogenous Vps1. Images are representative of three independent experiments.

To analyse localisation of Vps1 to peroxisomes, we expressed Vps1–GFP from a plasmid in *vps1Δ/dnm1Δ* cells controlled by its own promoter. Vps1–GFP rescued peroxisome fission (Fig. 3A), but no convincing colocalisation with peroxisomes was observed. The lack of Vps1–GFP localisation to peroxisomes might be a consequence of Vps1 being present briefly during a fission event as has been reported for the scission of endocytic vesicles from the plasma membrane (<10 s) (Smaczynska-de Rooji et al., 2010). To visualise Vps1 on peroxisomes, we used a GTPase-defective mutant (Vps1-K42A) that locks the protein in a constricted helical assembly on its membrane substrate (Sundborger et al., 2014; Tornabene et al., 2020; Varlakhanova et al., 2018). This mutant does not restore peroxisome fission in *vps1Δ/dnm1Δ* cells (Fig. 3A,B). Vps1-K42A–GFP is mainly localised to endosomal structures (Tornabene et al., 2020; Varlakhanova et al., 2018), but we also observed colocalisation of the GFP signal with peroxisomes (Fig. 3B). Vps1-K42A–GFP did not label the peroxisomal structure completely, but a punctate pattern was observed along the length of the elongated peroxisome (Fig. 3B). In *vps1Δ/dnm1Δ/pex27Δ* cells, Vps1-K42A–GFP no longer decorated the elongated peroxisomes (Fig. 3C). This suggests that Pex27 plays a specific role in Vps1 recruitment or assembly onto peroxisomal membranes. A Pex27–TAP-expressing strain (Ghaemmaghami et al., 2003) was transformed with a centromeric plasmid encoding Vps1–GFP under control of its endogenous promoter and, as negative control, a plasmid encoding GFP–PTS1 under control of the strong constitutive *TPI1* promoter. Using GFP–nanobody beads (GFP-Trap, ChromoTek), GFP–PTS1 and Vps1–GFP were precipitated. Pex27–TAP and endogenous Vps1 co-precipitated with Vps1–GFP but not with GFP–PTS1. This indicates that Pex27 and Vps1 can physically interact *in vivo* and that Vps1–GFP assembles into Vps1 oligomers (Fig. 3D).

### Pex27 localises to punctate structures along the peroxisomal membrane in *vps1Δ/dnm1Δ* cells

**Fig. 4.**
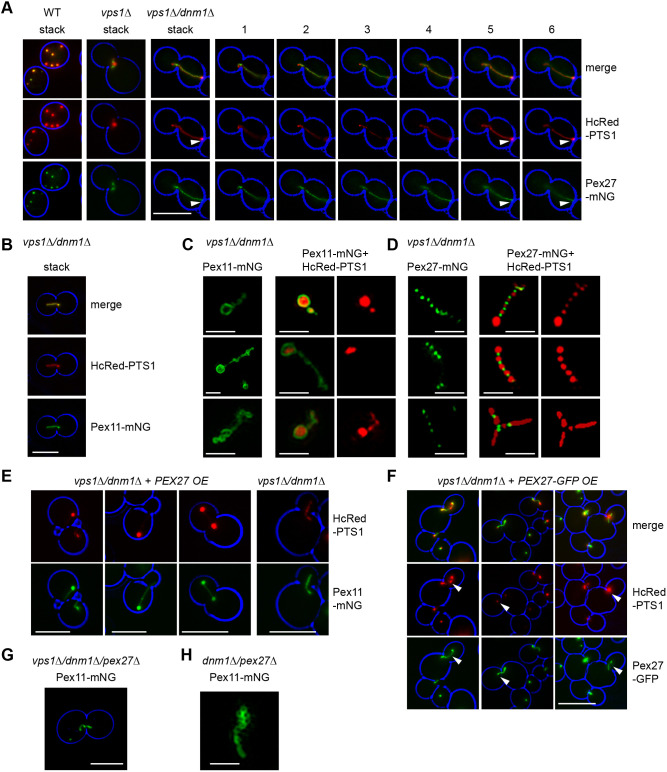
**Pex27–mNG is unequally distributed along the peroxisomal membrane.** (A,B) Representative epifluorescence microscopy images captured from WT, *vps1Δ* and *vps1Δ*/*dnm1Δ* cells co-expressing either Pex27–mNG or Pex11–mNG and HcRed–PTS1. Pex27 does not appear to label peroxisomes homogeneously in mutants with enlarged peroxisomes. ‘Stack’ represents flattened *z*-stacks; the numbers 1–6 indicate individual slices 0.5 µm apart. Arrowheads indicate low Pex27–mNG signal compared to that of HcRed–PTS1 at bulbous ends of the elongated peroxisome. (B) Pex11–mNG labels the peroxisomal membrane homogeneously in *vps1Δ*/*dnm1Δ* cells. (C,D) Analysis of the *vps1Δ*/*dnm1Δ* strains described in A and B with SIM. Both single-labelled examples and double-labelled examples are shown. Pex27 localises to a punctate pattern on the peroxisomal membrane between areas enriched with the matrix marker HcRed–PTS1. (E) *vps1Δ*/*dnm1Δ* cells overexpressing untagged Pex27 (*PEX27* OE) display membrane tubules weakly labelled with matrix (HcRed–PTS1) and membrane (Pex11–mNG) markers. (F) *vps1Δ*/*dnm1Δ* cells overexpressing Pex27–GFP controlled by the *TPI1* promoter (*PEX27-GFP* OE). Arrowheads indicate examples of HcRed–PTS1-labelled parts of elongated peroxisomes that are devoid of the Pex27–GFP label. (G,H) Pex27 is not essential for peroxisome constriction. Epifluorescence microscopy (G) and SIM (H) images of *vps1Δ*/*dnm1Δ/pex27Δ* (G) and *dnm1Δ/pex27Δ* (H) cells expressing Pex11–mNG from their endogenous locus, respectively. For A,B,E–G, the cell circumference is labelled in blue. Cells were grown for extended periods in log phase on 2% glucose-containing medium. Images are representative of three independent experiments. Scale bars: 5 μm (A,B,E–G); 1 μm (C,D,H).

We C-terminally tagged Pex27 with mNG at its endogenous genomic locus. In WT cells, Pex27–mNG localised to peroxisomes (Fig. 4A). Interestingly, Pex27–mNG did not label the complete peroxisome in mutants with enlarged tubular peroxisomes (*vps1Δ* and *vps1Δ/dnm1Δ* cells) as it appeared to be absent from the bulbous parts containing matrix proteins (Fig. 4A). This is in contrast to Pex11–mNG, which showed a complete overlap with the HcRed–PTS1 marker in *vps1Δ/dnm1Δ* cells (Fig. 4B). In *vps1Δ* and *vps1Δ/dnm1Δ* cells, peroxisomes form single elongated peroxisomes that consist of a chain of small peroxisomes connected via short constrictions (Hoepfner et al., 2001; Kuravi et al., 2006). As the resolution of epifluorescence microscopy is too low to clearly document sub-peroxisomal protein distribution, we resorted to structured illumination microscopy (SIM) using Pex11–mNG as the membrane marker and HcRed–PTS1 as the peroxisomal matrix marker. As expected, Pex11–mNG labelled the membrane of vesicles that were part of a single structure. The vesicle lumen was labelled with HcRed–PTS1 (Fig. 4C). In contrast, Pex27–mNG displayed a string of puncta. These puncta were present between puncta of the matrix marker (Fig. 4D). This indicates that Pex27 accumulates at sites of membrane constriction. Upon overexpression of untagged Pex27 in *vps1Δ/dnm1Δ* cells, peroxisome morphology changed from a tubular structure that was labelled throughout with both the matrix and membrane markers to either bulbous peroxisomes with very weakly labelled long extensions or dumbbell-shaped peroxisomes with very weakly labelled connecting tubules (Fig. 4E). These elongated tubules were absent in *vps1Δ/dnm1Δ/pex25Δ* cells overexpressing Pex27 (Fig. S3I), although some short extensions were observed in a low percentage of cells (<2% of peroxisome-containing cells) (Fig. S3I). Upon overexpression of Pex27–GFP in *vps1Δ/dnm1Δ* cells, the tubular extensions between the bulbous part of the peroxisomes were labelled with Pex27–GFP, whereas the bulbous parts were devoid of Pex27–GFP (Fig. 4F). The tubular connections between the bulbous parts again showed very weak luminal staining. Although the overexpression of Pex27 appeared to induce or extend narrow peroxisomal membrane tubules, peroxisomal membrane structures in *vps1Δ/dnm1Δ*/*pex27Δ* cells still showed constricted areas (Fig. 4G,H), indicating that membrane constriction does not require Pex27. In the few *vps1Δ/dnm1Δ/pex25Δ* cells overexpressing Pex27–GFP that contained peroxisomes with short extensions, Pex27–GFP was concentrated on the tubular part of these peroxisomes (Fig. S3J). This indicates that Pex27 does not require Pex25 for association with tubular parts of the peroxisomal membrane.

### Atg36 is not required for Vps1-dependent peroxisome multiplication in proliferating cells

**Fig. 5.**
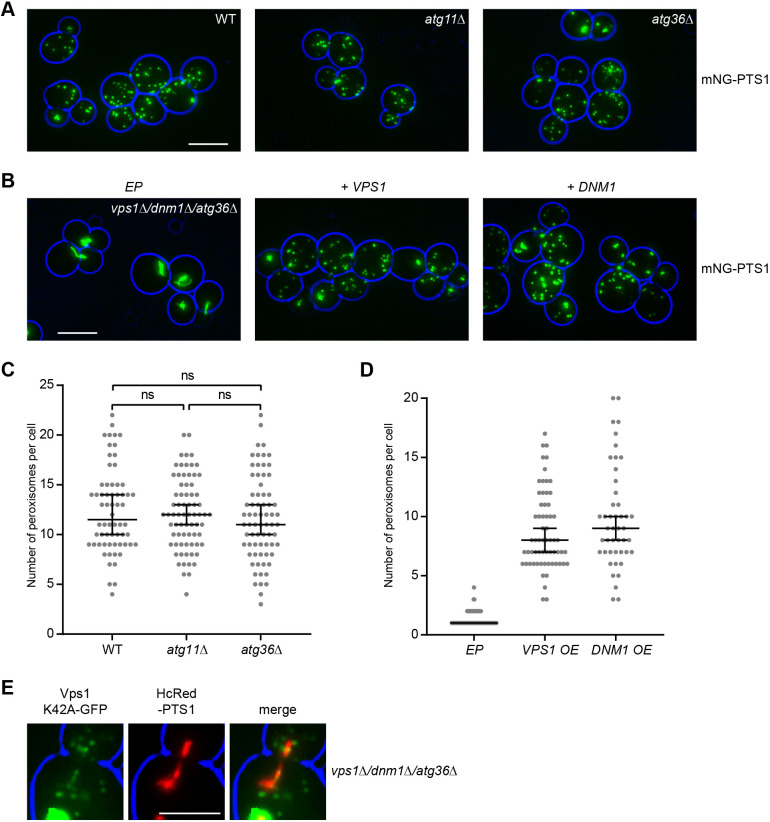
**Atg36 is not required for Vps1-dependent peroxisome multiplication in proliferating cells.** (A,B) Representative epifluorescence microscopy images captured from WT, *atg11Δ*, *atg36Δ* and *vps1Δ*/*dnm1/atg36Δ* cells expressing mNG–PTS1 that were grown for >24 h in log phase on 2% glucose-containing medium. Reintroduction of either *VPS1* or *DNM1* increases peroxisome number in *vps1Δ*/*dnm1/atg36Δ* cells. Scale bar: 5 μm. (C,D) Quantitation of A and B, respectively. Peroxisomes from budding cells were quantified and statistical variance was assessed using Kruskal–Wallis test. Bars in the graph indicate the median with 95% confidence intervals for mean. ns, not significant. (E) Epifluorescence microscopy images of *vps1Δ*/*dnm1/atg36Δ* cells expressing Vps1-K42A–GFP and HcRed–PTS1. Scale bar: 2.5 μm. For A,B,E, the cell circumference is labelled in blue. Images are representative of three independent experiments.

During starvation, peroxisomes are degraded by pexophagy. Efficient incorporation into pexophagophores requires peroxisomes to be divided by Vps1. Vps1 recruitment to the pexphagophore requires the pexophagy receptor Atg36 and the adapter Atg11 (Liu et al., 2018; Mao et al., 2014). However, peroxisome abundance in proliferating *atg36Δ* and *atg11Δ* cells is unaffected (Motley et al., 2012b) (Fig. 5A,C), suggesting that Vps1-dependent peroxisome fission under this condition does not require Atg36. To test this more directly, we generated an *ATG36*-deficient strain that is also blocked in Drp-dependent peroxisome fission (*vps1Δ/dnm1Δ/atg36Δ*) and reintroduced either Vps1 or Dnm1. Expression of either Vps1 or Dnm1 increased peroxisome number in this strain, indicating that Vps1 and Dnm1 were able to divide peroxisomes independent of Atg36 (Fig. 5B,D). Moreover, the localisation of Vps1-K42A to peroxisomes in proliferating *vps1Δ/dnm1Δ/atg36Δ* cells was not affected (Fig. 5E).

### Pex27 is not required for efficient pexophagy

**Fig. 6.**
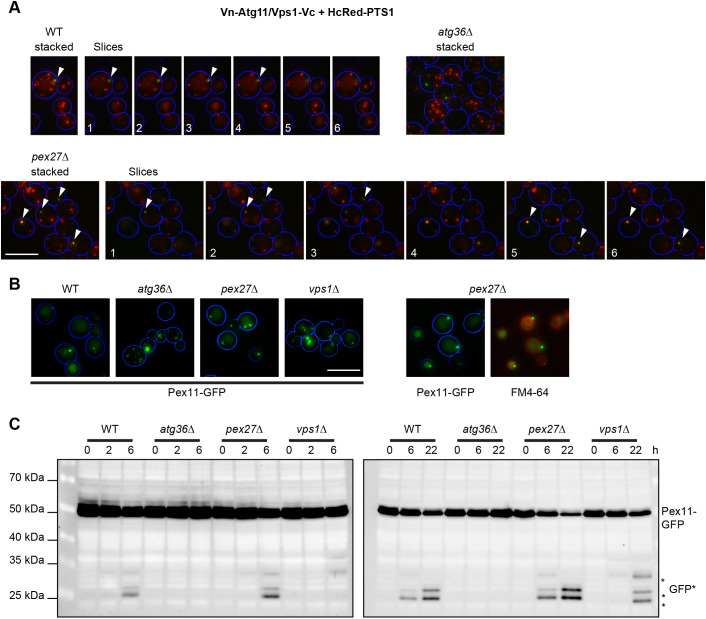
**Pex27 is not required for recruitment of Vps1 to the pexophagophore and efficient pexophagy.** (A) Epifluorescence micrograph of bimolecular fluorescence complementation of Vn–Atg11 and Vps1–Vc in WT, *atg36Δ and pex27Δ* cells expressing HcRed–PTS1. ‘Stacked’ represents flattened *z*-stacks; the numbers indicate individual slices 0.5 µm apart. Cells were grown overnight on oleate-containing medium and starved for 6 h on 2% glucose-containing medium lacking nitrogen. Arrowheads indicate the proximal localisation of the Venus signal and the peroxisomal marker. (B) Representative epifluorescence microscopy images captured from WT, *atg36Δ*, *pex27Δ* and *vps1Δ* cells expressing Pex11–GFP (left). Cells were grown overnight on oleate-containing medium and starved for 22 h on 2% glucose-containing medium lacking nitrogen. *pex27Δ* cells were subsequently stained with FM4-64 to visualise the vacuolar membrane (right). The cell circumference is labelled in blue. Scale bars: 5 μm. (C) The initiation and level of pexophagy was assessed by western blotting for Pex11–GFP breakdown using anti-GFP at different time points in the respective mutant strains. Pexophagy was induced as in A. Asterisks indicate Pex11–GFP breakdown products. Images are representative of three independent experiments.

Recruitment of Vps1 to the pexophagophore via the Atg11 and Atg36 complex was previously visualised by bimolecular fluorescence complementation (Mao et al., 2014). Indeed, in cells co-expressing Vps1–Vc and Vn–Atg11 (Vc and Vn are the C- and N-terminal parts of the Venus fluorescent protein, respectively), a clear Venus signal was observed in the proximity of peroxisomes (Fig. 6A). Although a signal was observed in *atg36Δ* cells, this signal did not localise to peroxisomes. Using this assay, we found that Vps1 recruitment to the pexophagophore was unaffected by deletion of *PEX27* (Fig. 6A). To test the efficiency of pexophagy in *pex27Δ* cells, we analysed cells expressing Pex11–GFP using fluorescence microscopy (Fig. 6B) and the accumulation of a Pex11–GFP cleavage product that forms upon entry to vacuoles as semi-quantitative measures of pexophagy (Fig. 6C) (Motley et al., 2012b). This analysis revealed that in contrast to *vps1Δ* cells, *pex27Δ* cells were unaffected in the timing of initiation and the level of pexophagy. We conclude that Pex27 is not required for pexophagy.

## DISCUSSION

**Fig. 7.**
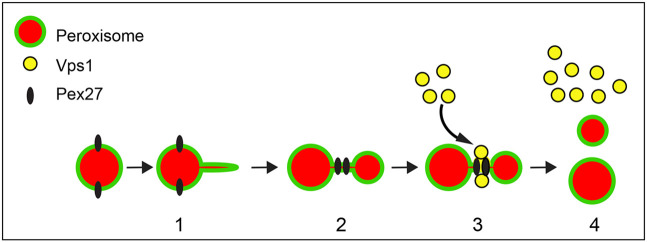
**Working model for the requirement of Pex25 and Pex27 in Vps1-dependent peroxisome fission.** The multistep process of peroxisome fission is initiated by (1) Pex25-dependent protrusion and elongation of the peroxisomal membrane. (2) Import of proteins allows peroxisomes to grow and Pex27 concentrates on the highly curved membrane at tubular, constricted regions between bulbous areas. (3) Vps1 associates with peroxisomes dependent on Pex27. Vps1 forms helical oligomeric assemblies around the membrane tube and GTP hydrolysis induces a conformational change and constriction of the membrane which leads to (4) fission and Vps1 disassembly.

The dynamin-related protein Vps1 requires auxiliary factors for its recruitment and activity during a variety of membrane remodelling processes. Here, we report that Vps1-dependent peroxisome fission, but not Dnm1-dependent peroxisome fission, requires the peroxisomal membrane protein Pex27 and that Pex27 localises to constricted areas of peroxisomes when fission is blocked. In addition, we show that Pex27 and Vps1 are able to physically interact *in vivo* and that an increase in the level of Pex27 increases peroxisome number in a manner dependent upon Vps1. A GTPase mutant of Vps1, Vps1-K42A, that mainly localises to endosomes (Sundborger et al., 2014; Tornabene et al., 2020; Varlakhanova et al., 2018) also associates with peroxisomes and this association depends on Pex27. These observations support a model wherein Pex27 acts as a specific Vps1 cofactor on the peroxisomal membrane (Fig. 7). Interestingly, Pex27 is not required during Vps1-dependent fission of peroxisomes during pexophagy.

Peroxisome multiplication is a multistep process during which peroxisomes generate a membrane protrusion that subsequently elongates and starts importing matrix proteins. Subsequently, dynamin-related proteins divide the peroxisomes at constricted areas between the bulbous parts (see review by Schrader et al., 2016). In mammals and the yeast *H. polymorpha*, the tubulation of the peroxisomal membrane is induced by Pex11. Membrane remodelling by Pex11 is coupled to recruitment of fission factors (Fis1, Drp1 and, in mammals, MFF) to sites of membrane constriction (Imoto et al., 2020; Williams et al., 2015). Pex11 also acts at the scission stage as Pex11 interacts directly with Drp1 and stimulates its GTPase activity *in vitro*. Mutants that block this interaction block *in vitro* GTPase activation and peroxisome fission *in vivo* (Williams et al., 2015).

In *S. cerevisiae*, Dnm1 and Pex11 play a minor role in peroxisome multiplication that is most obvious under conditions of peroxisome proliferation (Erdmann and Blobel, 1995; Kuravi et al., 2006; Motley et al., 2008) (see also Fig. S1). In contrast, Vps1-, Pex25- and Pex27-deficient cells display a strong reduction of peroxisome number, especially in rapidly dividing cells. Pex25 plays a crucial role in the generation of the initial protrusion and elongation of peroxisomal membrane tubules (Huber et al., 2012) and, therefore, resembles Pex11. Elongated tubular peroxisomes characteristic of *vps1Δ/dnm1Δ* cells are indeed mostly absent in *vps1Δ/dnm1Δ/pex25Δ* cells (Fig. 2D; Figs S1, S3I). Detailed mechanistic studies of Pex25 have not been reported but, like Pex11β, Pex25 contains a predicted amphipathic helix in its N-terminal half that might be required for membrane tubulation. The role of Pex25 in elongation is also unknown but it is tempting to speculate that like *S. cerevisiae* Pex11, Pex25 is part of a membrane contact site and that the Pex25 membrane contact site allows membrane lipid flux into growing peroxisomal membrane tubules, analogous to the role of the endoplasmic reticulum–peroxisome tether ACBD4/5 and VAPB (Costello et al., 2017a,b; Hua et al., 2017). Pex25 is required for both Vps1-dependent and Dnm1-dependent peroxisome fission. Even overexpression of these Drps cannot induce peroxisomes to divide in the absence of Pex25. The Pex25 paralogue, Pex27, is not required for elongation of peroxisomes (Fig. 1A), their tubulation or their constriction (Fig. 3C, Fig. 4G,H; Fig. S1). As Pex27 overexpression does not restore peroxisome number in *pex25Δ* cells, the two paralogues appear to have specific function(s). We identified Pex27 as a factor specifically required for Vps1-dependent peroxisome fission. It concentrates in constricted areas of the peroxisomal membrane in cells in which fission is blocked. This observation is further corroborated by experiments in which Pex27–GFP is overexpressed. Pex27 concentrates on the tubules connecting the bulbous parts (Fig. 4F). These tubules, however, are very dimly labelled with peroxisomal matrix proteins, suggesting that they are extended constrictions. Whereas Pex25 acts at early stages of peroxisome multiplication, in the protrusion and elongation stages, our data support a role for Pex27 in Vps1-dependent fission after constriction of the peroxisomal membrane. Its position at constriction sites places Pex27 ideally to either recruit Vps1 directly or to modify the constriction site, through, for instance, remodelling of the membrane or recruitment of other factors, to allow local assembly of Vps1 oligomers. These options are in line with our observation that the Vps1-K42A mutant accumulates on peroxisomes dependent on Pex27. Vps1-K42A is a GTP hydrolysis-deficient mutant that forms helical assemblies in a hyper-constricted state, which fail to disassemble and therefore accumulate on target membranes (Tornabene et al., 2020; Varlakhanova et al., 2018). As we could not detect Vps1-K42A–GFP on peroxisomes in *vps1Δ/dnm1Δ/pex27Δ* cells, we conclude that Pex27 acts prior to Vps1 reaching its hyper-constricted state on the peroxisomal membrane. We cannot exclude additional later roles for Pex27 in Vps1-dependent fission, for instance, in the regulation of Vps1 GTPase activity, analogous to the functions of *H. polymorpha* Pex11 and human Pex11β in the regulation of Drp1 (Williams et al., 2015).

In contrast, previous overexpression studies with *PEX27* were interpreted to counteract Pex25 function in peroxisome multiplication (Huber et al., 2012). This conclusion was based on the observation that Pex27 overexpression resulted in a partial mislocalisation of a peroxisomal matrix marker to the cytosol and decreased growth on oleate-containing medium. We confirmed that Pex27 overexpression induces a partial block in matrix protein import (Fig. S3A). However, when using peroxisomal membrane markers, we found that Pex27 overexpression induced extensive Vps1-dependent peroxisome fission and we therefore conclude that Pex27 does not counteract Pex25 in peroxisome fission. Why Pex27 overexpression induces mislocalisation of matrix proteins is not clear, but it is unrelated to the extensive fission of peroxisomes as even in cells lacking Vps1, matrix proteins were mislocalised (Fig. S3A).

Vps1 has been reported to divide larger peroxisomes to accommodate their engulfment by autophagosomal membranes and promote efficient pexophagy. Vps1 is recruited to pexophagophores via the Atg36/Atg11 pexophagy receptor complex (Mao et al., 2014). We found that neither Atg36 nor Atg11 was required for peroxisome multiplication during exponential growth and for the association of Vps1-K42A with peroxisomes. Neither did we find a requirement for Pex27 in the recruitment of Vps1 to the pexophagophore or for pexophagy.

Taken together, our results expand the set of factors that allow Vps1 to act in various membrane remodelling processes and we conclude that Vps1 function in peroxisome maintenance under different growth conditions is aided by process-specific auxiliary factors.

## MATERIALS AND METHODS

### Strains and plasmids

*S. cerevisiae* strains used in this study are shown in Table S1. Yeast strains were derivatives of either BY4741 (*MATA his3Δ1 leu2Δ0 met15Δ0 ura3Δ0*) or BY4742 (*MATα his3Δ1 leu2Δ0 lys2Δ0 ura3Δ0*) obtained from the EUROSCARF consortium. Double or triple gene deletions were made by replacing the entire coding sequence of the desired gene with either *Schizosaccharomyces pombe HIS5* or *Klebsiella pneumoniae* hygromycin B phosphotransferase that confers resistance to hygromycin B (Goldstein and McCusker, 1999). The pFA6a-yomNeonGreen (mNG)-spHIS5 plasmid was used as a template for PCR to tag Pex11 and Pex27 at the C-terminal with mNG (Shaner et al., 2013).

The plasmids used in this study are listed in Table S2. *URA3* and *LEU2* centromeric plasmids were derived from Ycplac33 and Ycplac111 (Gietz and Sugino, 1988) and contained the *PGK1* terminator. These ARS1/CEN4 plasmids are present at one to two copies per cell (Falcon and Aris, 2003). The plasmid constructs were generated either by the gap repair mechanism in yeast (Orr-Weaver and Szostak, 1983) or by conventional restriction digestion-ligation-based methods in *Escherichia coli* (Cohen et al., 1973). Constitutive expression of HcRed–PTS1, mNG–PTS1, mKate2–PTS1 and GFP was under either the *HIS3* or *TPI1* promoter and the conditional expression plasmids contained the *GAL1* promoter. *DNM1* and *VPS1* overexpression was achieved using the *TPI1* promoter and was described previously (Motley et al., 2008). Expression of Vps1–GFP and Vps1-K42A–GFP was achieved through the Vps1 promoter and Pex27–ProtA was under control of its own promoter.

### Growth conditions

For the screen presented in Fig. S1, cells were grown overnight in a defined selective 2% glucose-containing medium at 30°C. For analysis of phenotypes by microscopy, cells were subsequently diluted to an optical density at 600 nm (OD_600_) of 0.1 in a fresh selective 2% glucose-containing medium and grown for at least three cell divisions (6 h) prior to imaging. Certain phenotypes are sensitive to cell growth rate. For instance, peroxisome inheritance defects are compensated for by *de novo* formation and the number of cells without peroxisomes increases in exponentially growing cultures versus stationary-phase cultures (Hettema and Motley, 2009). Likewise, the peroxisome number in pexophagy mutants is affected by growth rate (Nuttall et al., 2014). Therefore, in subsequent experiments, we made sure that overnight culture did not reach the stationary phase before they were diluted to an OD_600_ of 0.1 in the morning. Where the induction of a reporter protein was required, cells were transferred to a selective galactose medium at an OD_600_ of 0.1 and grown for the time indicated in the figures and text. Yeast cells were grown at 30°C in either of the following mediums: rich YPD medium (1% yeast extract, 2% peptone, 2% glucose), minimal medium 2 (YM2) for the selection of the uracil prototrophic marker (carbon source, 0.17% yeast nitrogen base without amino acids and ammonium sulphate, 0.5% ammonium sulphate, 1% casamino acids) or minimal medium 1 (YM1) for the selection of all prototrophic markers (carbon source, 0.17% yeast nitrogen base without amino acids and ammonium sulphate, 0.5% ammonium sulphate). As carbon sources, 2% (w/v) glucose and galactose were added. For induction of peroxisome proliferation, cells were transferred to oleate-containing medium [YM2 oleate: YM2 plus 0.12% oleate (v/v), 0.2% Tween-40s (v/v), 0.1% yeast extract] at a 1/10 overnight dilution. Pexophagy was induced by transferring cells to starvation medium lacking a nitrogen source (SD-N; 0.17% yeast nitrogen base without amino acids and ammonium sulphate, 2% glucose) (Hutchins et al., 1999; Lynch-Day and Klionsky, 2010). The appropriate amino acid stocks were added to minimal medium as required. In all, ten OD_600_ units were collected at the selected time points as indicated in the figures and text. Cells were either analysed by immunoblotting or by fluorescence microscopy. For peroxisome quantification, the budding cells were considered as single cells. Mating experiments were performed as described previously (Motley and Hettema, 2007). Briefly, for mating, one OD_600_ unit of MATα cells were first induced with galactose for 3 h and subsequently chased for 2 h on YPD, before they were mixed with one OD_600_ unit of MATA cells, pelleted, spotted onto a prewarmed YPD plate and incubated at 30°C for 2 h before imaging. The vacuolar membrane was stained with FM4-64 (Invitrogen, T3166) as previously described (Vida and Emr, 1995).

### Image acquisition

Cells were analysed with a microscope (Axiovert 200M; Carl Zeiss) equipped with an Exfo X-cite 120 excitation light source, band pass filters (Carl Zeiss and Chroma Technology), an α Plan-Fluar 100×1.45 NA and Plan-Apochromat 63×1.4 NA objective lens (Carl Zeiss) and a digital camera (Orca ER; Hamamatsu Photonics). Image acquisition was performed using Volocity software (PerkinElmer). Fluorescence images were collected as 0.25 or 0.5 μm *z*-stacks, merged into one plane using Openlab software (PerkinElmer) and processed further in Photoshop (Adobe). Bright-field images were collected in one plane and processed where necessary to highlight the circumference of the cells in blue. Each imaging experiment was performed at least three times, and representative images are shown. For quantitation, a single experiment was used. For localisation of Pex11–mNG and Pex27–mNG *in vivo*, cells were imaged with DeltaVision/GE OMX optical microscope equipped with laser lines (488 nm and 568 nm) and 60×1.42 NA oil Plan-Apochromat to perform SIM. Image acquisition was performed using DeltaVision OMX SoftWoRx 6.0 software. Fluorescence images were collected as 0.25 μm *z*-stacks, merged into one plane in Fiji (Schindelin et al., 2012) and processed further in Adobe Photoshop. To immobilise cells, a 2% agarose gel pad containing minimal growth medium was prepared into a glass-bottomed 35 mm μ-dish (ibidi). The cells were grown logarithmically and 20 μl culture was supplied under the gel pad and spread uniformly by gently pressing the gel pad from the top.

### Immunoblotting

For preparation of extracts by alkaline lysis, cells were centrifuged, and pellets resuspended in 0.2 M NaOH and 0.2% β-mercaptoethanol and left on ice for 10 min. The soluble protein was precipitated by addition of 5% trichloroacetic acid and incubation on ice for further 15 min. Following centrifugation (13,000 ***g***, 5 min, 4°C), the pellet was resuspended in 10 μl 1 M Tris-HCl (pH 9.4) and 90 μl 1× SDS–PAGE sample loading buffer, and boiled for 10 min at 95°C. Samples (0.25–1 OD_600_ equivalent) were resolved by SDS–PAGE followed by immunoblotting. Blots were blocked in 2% (w/v) fat-free Marvel milk in TBS containing Tween-20 [50 mM Tris-HCl pH 7.6, 150 mM NaCl, 0.1% (v/v) Tween-20]. GFP-tagged proteins were detected using a monoclonal anti-GFP antibody (mouse IgG monoclonal antibody clone 7.1 and 13.1; 1:3000; Roche, 11814460001). Pex27–ProtA and Pex27–TAP were detected by the peroxidase-anti-peroxidase (PAP) antibody (rabbit; 1:4000; Sigma-Aldrich, P1291). Vps1 was detected with the polyclonal anti-Vps1 antibody (rat; 1:10,000; gift by Kathryn Ayscough, School of Biosciences, University of Sheffield, UK). The specificity of this antiserum is shown by the lack of a signal in *vps1Δ* cells in Fig. 1C. Pgk1 was detected by monoclonal anti-Pgk1 antibody (mouse; 1:7000; Invitrogen, 459250). The secondary antibody was a HRP-linked anti-mouse polyclonal (goat; 1:4000; Bio-Rad, 1706516) or HRP-linked anti-rat polyclonal (rabbit; 1:10,000, Sigma-Aldrich, A5795) antibody. Detection was achieved using enhanced chemiluminescence reagents (GE Healthcare) and chemiluminescence imaging.

### Coimmunoprecipitation

For immunoprecipitation experiments, we transformed Pex27–TAP-expressing cells (Ghaemmaghami et al., 2003) with a centromeric plasmid encoding GFP–PTS1 under control of the *TPI1* promoter (pEH012) or Vps1–GFP under control of its endogenous promoter (pKA1078, Ayscough laboratory) or an empty plasmid (Ycplac33). Logarithmically growing cells at an OD_600_ of 50–60 were harvested and washed once with 50 mM HEPES-KOH, pH 7.6, before freezing at −80°C. The cell pellet was thawed and resuspended in 600 μl of ice-cold lysis buffer (50 mM HEPES-KOH, pH 7.6, 150 mM KCl, 100 mM β-glycerol phosphate, 25 mM NaF, 1 mM EGTA, 1 mM MgCl_2_, 0.15% Tween-20, protease inhibitor cocktail). Subsequently, 400 μl of acid-washed glass beads (Merck, G9268) were added to the above mixture. The cells were lysed by means of a glass bead beater (Mini-BeadBeater-16, Glen Mills) for two 30 s rounds at top speed and 2 min on ice after each round. The tubes were centrifuged for 5 min at 15,000 ***g*** at 4°C. Approximately 400 μl of supernatant was collected and replaced with 400 μl of lysis buffer and the samples were beaten and centrifuged again as mentioned above. The supernatants were pooled together and further cleared by centrifugation (5 min at 15,000 ***g***, 4°C). The clear supernatant was transferred to the affinity-purification beads (GFP-Trap Agarose, GTA, ChromoTek) pre-equilibrated in the lysis buffer. From the cell lysate samples, 45 μl was taken before treatment with affinity beads as the input material. The tubes were incubated on a rotating wheel at 4°C for 2 h and then washed three times with the lysis buffer supplemented with 10% glycerol and no protease inhibitors. Then, the beads were transferred to fresh tubes and washed once more before adding 100 μl 1× protein loading dye. The samples were boiled at 95°C for 10 min and analysed by western blotting. GFP fusions were detected using anti-GFP, endogenous Vps1 was detected with anti-Vps1 and Pex27–TAP was detected with PAP. For further details see the ‘Immunoblotting’ section above.

## Supplementary Material

10.1242/joces.246348_sup1 Supplementary information
