# Supplementary material for: The dynamin-related protein Vps1 and the peroxisomal membrane protein Pex27 function together during peroxisome fission
Source: J Cell Sci. 2023 Mar 24;136(6):jcs246348. doi: 10.1242/jcs.246348 (PMC10112978; doi:10.1242/jcs.246348)
Supplement: Supplementary information [file joces-136-246348-s1.pdf]

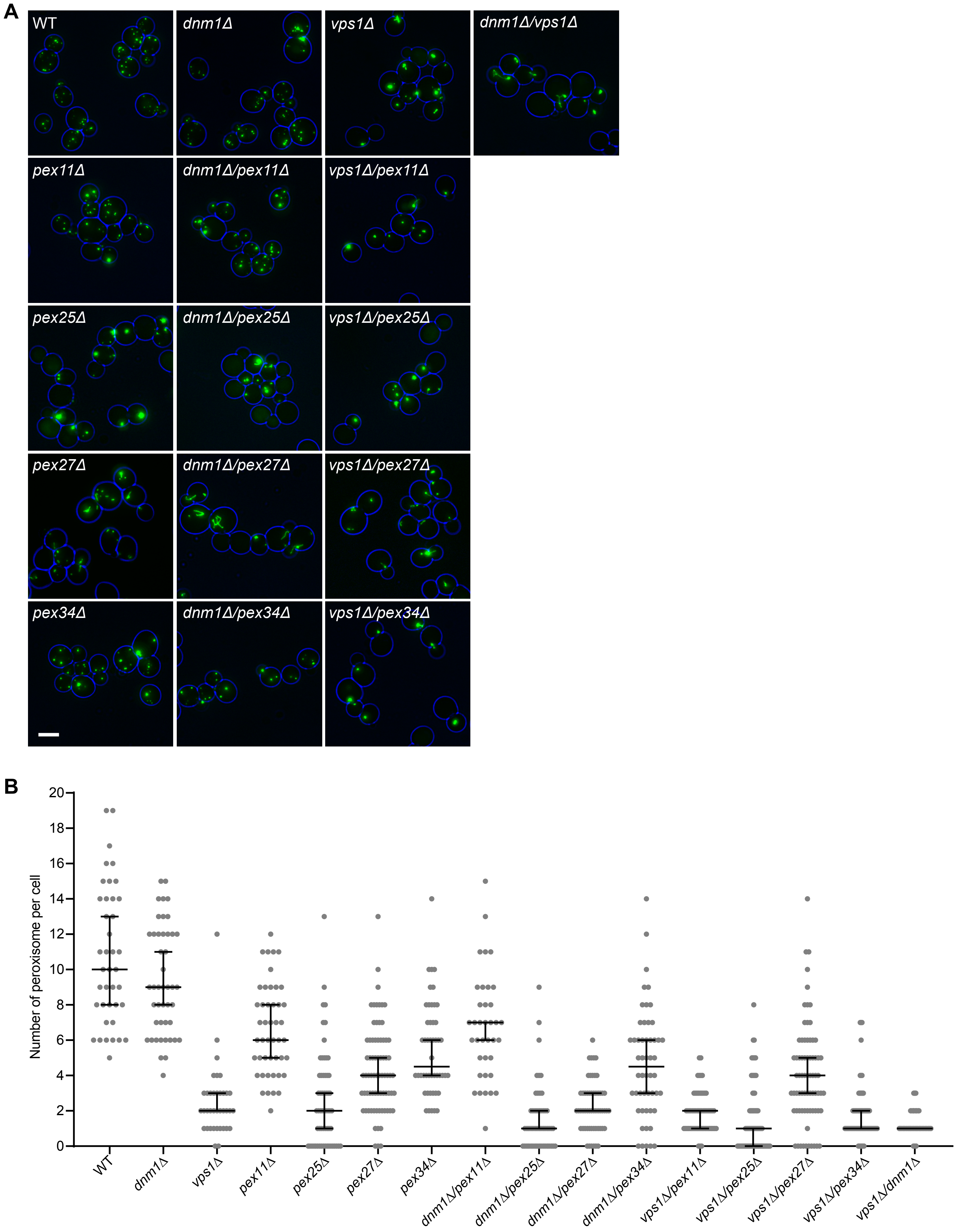

Fig. S1. Targeted screen for peroxisome multiplication: (A) Epifluorescence images captured from the various *Saccharomyces cerevisiae* mutant cells expressing mNeonGreen fluorescent protein appended with a peroxisomal targeting signal type I (mNG-PTS1). Cells were grown for an extended period on 2% glucose containing media. Representative images are shown as merged Z-stacks. Cell circumference is labelled blue. Scale bar, 5 μm. (B) Graph showing the distribution of peroxisome abundance for the strains indicated in (A). A minimum of 37 budding cells were analysed. Error bars represent the median with 95% confidence interval.

Journal of Cell Science • Supplementary information

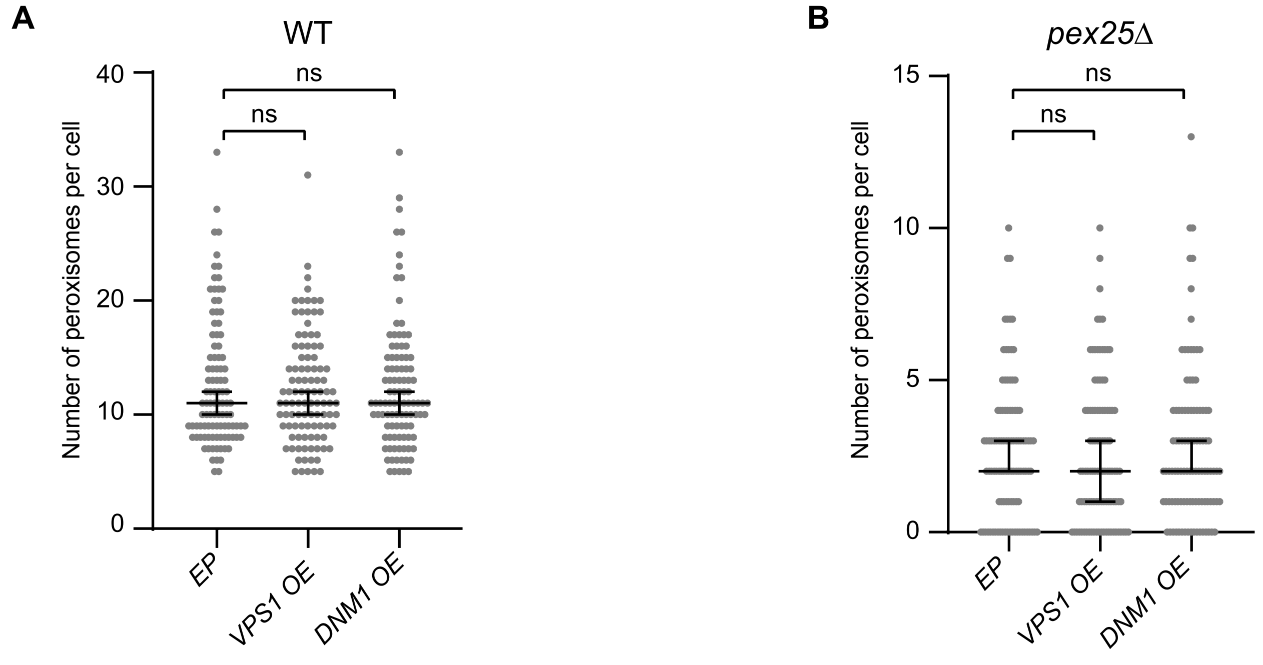

Fig. S2. *VPS1* and *DNM1* overexpression does not increase peroxisome abundance in WT and *pex25Δ* cells. WT (A) and *pex25Δ* (B) cells expressing mNG-PTS1 were transformed with either *VPS1* or *DNM1* under the control of the strong constitutive *TPI1* promoter to induce *VPS1* overexpressing (*VPS1 OE*) and *DNM1* overexpression (*DNM1 OE*) or empty plasmid as negative control (EP). Cells from exponential growing cultures were imaged using epifluorescence microscopy and the distribution of peroxisome abundance was determined. Statistical significance analysis was performed using the Kruskal-Wallis test, ns: no statistical significant difference was observed.

Journal of Cell Science • Supplementary information

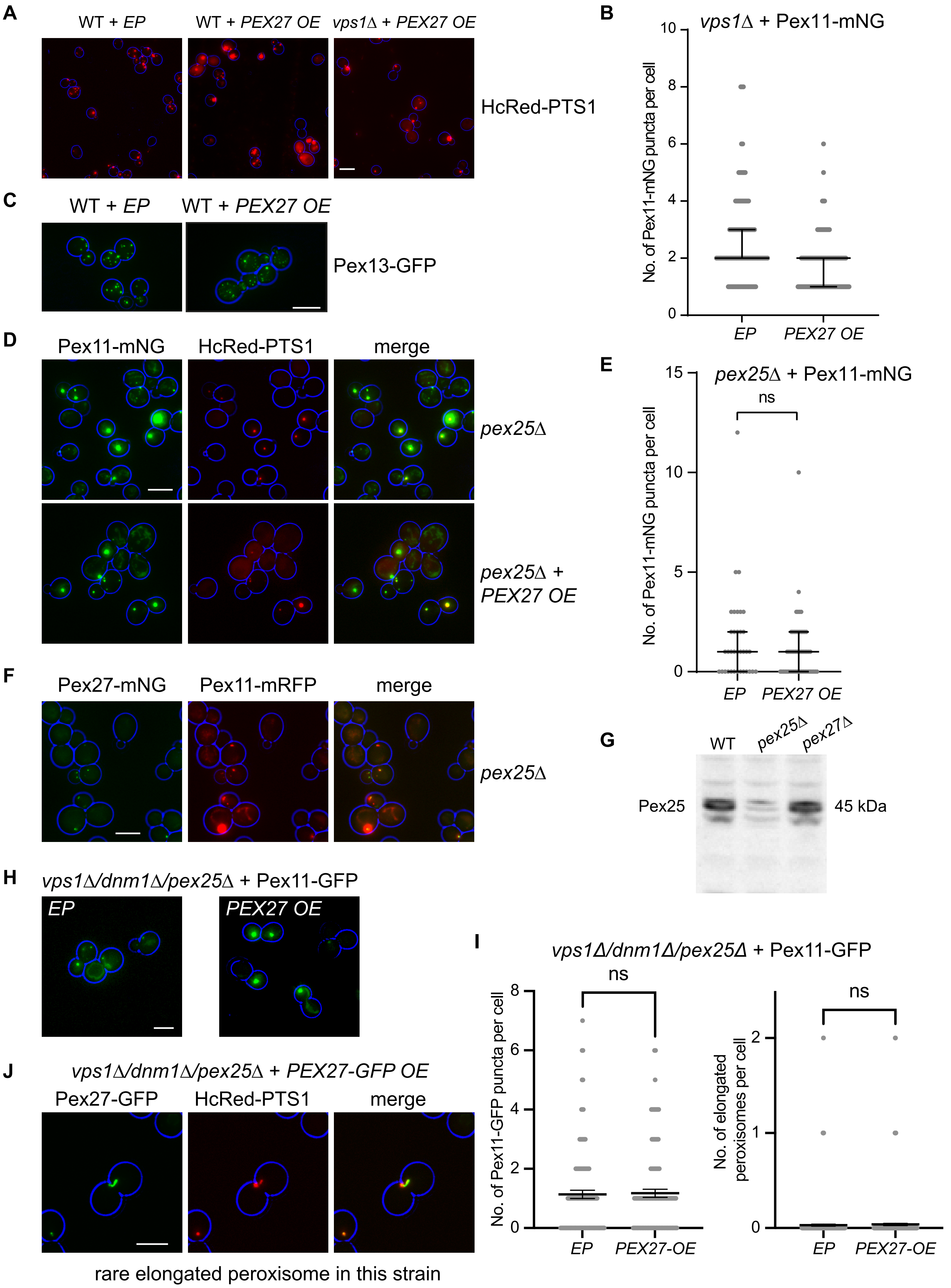

Journal of Cell Science • Supplementary information

Fig. S3. *PEX27* overexpression induces multiplication of peroxisomes dependent on Vps1 and Pex25. (A) *PEX27* overexpression induces partial mislocalisation of the matrix protein marker HcRed-PTS1. WT and *vps1Δ* cells expressing HcRed-PTS1 were transformed with either an empty plasmid (EP, negative control) or *PEX27* under the control of the strong constitutive *TPI1* promoter to induce *PEX27* overexpression (*PEX27 OE*). Note partial mislocalisation of HcRed-PTS1 to cytosol in both WT and *vps1Δ* cells upon *PEX27* overexpression. (B) In *vps1Δ* cells expressing Pex11-mNG from its endogenous locus, *PEX27* overexpression does not increase mNG-labelled peroxisomal membrane structures (see also the main manuscript Fig. 2B,C). (C) *PEX27* overexpression induces an increase in Pex13-GFP labelled structures in WT cells. (D,E) Epifluorescence analysis of *pex25Δ* cells expressing Pex11-mNG from its endogenous locus and HcRed-PTS1 from a plasmid. Note how cells lacking HcRed-PTS1 puncta mislocalise Pex11-mNG to a tubular network. Overexpression of *PEX27* does not affect the Pex11-mNG labelling pattern in *pex25Δ* cells (D) or the abundance of Pex11-mNG structures (E).(F) Pex27-mNG expressed from its endogenous locus colocalises with Pex11-mRFP in *pex25Δ* cells. (G) Western blot analysis of total cellular lysates of glucose grown cells using Pex25 antiserum. Pex25 expression level is unaffected in *pex27Δ* cells. (H,I) Epifluorescence microscopy analysis of *vps1Δ/dnm1Δ/pex25Δ* cells overexpressing *PEX27.* Pex27 overexpression does not affect the Pex11-mNG labelling pattern or abundance or shape in *vps1Δ/dnm1Δ/pex25Δ* cells. Note that *PEX27* overexpression does not induce tubulation of peroxisomes in this mutant, however in less than 2% of the cells we did observe short elongated peroxisomes. See (J), (J) In the rarely elongated peroxisomes observed in *vps1Δ/ dnm1Δ/pex25Δ* cells, Pex27-GFP localises to tubular parts of these peroxisomes. (B,E,I) Statistical significance analysis was performed using the Kruskal-Wallis test, ns: no statistical significant difference was observed. Cells from exponential growing cultures were used for all epifluorescence microscopy experiments. Scale bar, 5 μm. Cell circumference is labelled blue.

Journal of Cell Science • Supplementary information

Table S1. Yeast strains used in this study.

Strain and genotype	Reference
BY4741 MATA his3Δ1 leu2Δ0 met15Δ0 ura3Δ0	EUROSCARF
BY4742 MATα his3Δ1 leu2Δ0 lys2Δ0 ura3Δ0	EUROSCARF
BY4742 pex11Δ::kanMX4	EUROSCARF
BY4742 pex25Δ::kanMX4	EUROSCARF
BY4742 pex27Δ::kanMX4	EUROSCARF
BY4742 pex34::his3MX6	This study
BY4742 dnm1Δ::kanMX4	EUROSCARF
BY4742 vps1Δ::kanMX4	EUROSCARF
BY4742 dnm1Δ::kanMX4 vps1Δ::his3MX6	(Motley and Hettema, 2007)
BY4742 dnm1Δ::kanMX4 pex11Δ::his3MX6	This study
BY4742 dnm1Δ::kanMX4 pex25Δ::his3MX6	This study
BY4742 dnm1Δ::kanMX4 pex27Δ::his3MX6	This study
BY4742 dnm1Δ::kanMX4 pex34Δ::his3MX6	This study
BY4742 vps1Δ::kanMX4 pex11Δ::his3MX6	This study
BY4742 vps1Δ::kanMX4 pex25Δ::his3MX6	This study
BY4742 vps1Δ::kanMX4 pex27Δ::hphMX4	This study
BY4742 vps1Δ::kanMX4 pex34Δ::his3MX6	This study
BY4742 pex27Δ::kanMX4 pex25::hphMX4	This study
BY4741 dnm1Δ::kanMX4 vps1Δ::loxp pex25 Δ::hphMX4	This study
BY4742 dnm1Δ::kanMX4 vps1Δ::loxP pex27Δ::his3MX6	This study
BY4741 pex3Δ::kanMX4	EUROSCARF
BY4741 pex3Δ::kanMX4 vps1Δ::hphMX4	(Motley and Hettema, 2007)
BY4742 dnm1Δ::kanMX4 vps1Δ::loxP PEX11::PEX11-mNG-HIS3	This study
BY4742 dnm1Δ::kanMX4 vps1Δ::loxP PEX27::PEX27-mNG-HIS3	This study
BY4741 PEX27::PEX27-TAP-HIS3	(Ghaemmaghami et al., 2003)
BY4741 atg36Δ::KanMX4	(Motley et al., 2012b)
BY4742 atg11Δ:: KanMX4	(Motley et al., 2012b)
BY4742 dnm1Δ::kanMX4 vps1Δ::loxP atg36Δ::his3MX6	This study
SEY6210 RPL7Bp-VN-ATG11::TRP1	(Mao et al., 2013)
SEY6210 RPL7Bp-VN::TRP1	(Mao et al., 2014)
SEY6210 RPL7Bp-VN-ATG11::TRP1 atg36Δ::his3MX6	This study
SEY6210 RPL7Bp-VN-ATG11::TRP1 pex27Δ::his3MX6	This study
BY4742 pex25Δ::kanMX4 PEX11::PEX11-mNG-HIS3	This study
BY4742 pex25Δ::kanMX4 PEX27::PEX27-mNG-HIS3	This study
BY4742 pex25Δ::kanMX4 TRP1::mNG-PTS1-HIS3	This study

Journal of Cell Science • Supplementary information

Table S2. The plasmids used in this study.

Plasmid Name	Vector backbone	Promoter	Insert	Source
pAUL3	Ycplac33	*HIS3*	*mNG-PTS1*	Lab stock
pAUL4	Ycplac111	*HIS3*	*mNG-PTS1*	Lab stock
pLE140	Ycplac33	*PEX27*	*PEX27-ProtA*	This study
pGFP-Snc1	pRS416	*TPI1*	*GFP-Snc1*	(Lewis et al., 2000)
pAS5	Ycplac33	*HIS3*	*Hc-Red-PTS1*	Lab stock
pAS63	Ycplac111	*HIS3*	*Hc-Red-PTS1*	Lab stock
pAUL7	Ycplac111	*GAL1*	*mNG-PTS1*	Lab stock
pAUL28	Ycplac33	*HIS3*	*mKate2-PTS1*	Lab stock
pEW318	Ycplac33	-	-	Lab stock
pEW319	Ycplac111	-	-	Lab stock
pEH077	Ycplac111	*TPI1*	*3xHA-DNM1*	(Motley et al., 2008)
pEH079	Ycplac111	*TPI1*	*3xHA-VPS1*	(Motley et al., 2008)
pLE48	Ycplac111	*TPI1*	*PEX27*	This study
pKA1078	Ycplac33	*VPS1*	*VPS1-GFP*	Kathryn Ayscough
pLE141	Ycplac33	*VPS1*	*VPS1-K42A-GFP*	This study
pLE44	pFA6a	-	*mNG-HIS3*	This study
pEH012	Ycplac33	*TPI1*	*GFP-PTS1*	Lab stock
pLE41	Ycplac111	*TPI1*	*PEX27-GFP*	This study
	pRS416	*VPS1*	*VPS1-VC*	(Mao et al., 2014)
pEH007	Ycplac111	*PEX11*	*PEX11-GFP*	(Motley et al., 2012b)
pAS199	Ycplac111	*PEX11*	*PEX11-mRFP*	(Motley et al., 2015)
pEH101	Ycplac33	*PEX13*	*PEX13-GFP*	(Motley et al., 2015)

Journal of Cell Science • Supplementary information
