# Supplementary material for: The dynamin-related protein Vps1 and the peroxisomal membrane protein Pex27 function together during peroxisome fission
Source: J Cell Sci. 2023 Mar 24;136(6):jcs246348. doi: 10.1242/jcs.246348 (PMC10112978; doi:10.1242/jcs.246348)
Supplement: Supplementary file 1 [file joces-136-246348_review_history.pdf]

The dynamin-related protein Vps1 and the peroxisomal membrane protein Pex27 function together during peroxisome fission

Lakhan Ekal, Abdulaziz M. S. Alqahtani and Ewald Hettema
DOI: 10.1242/jcs.246348

Editor: Jennifer Lippincott-Schwartz

Review timeline
Original submission: 16 March 2020
Editorial decision: 21 May 2020
First revision received: 2 October 2022
Accepted: 10 February 2023

Original submission

First decision letter

MS ID#: JOCES/2020/246348

MS TITLE: The dynamin-related protein Vps1 and the peroxisomal membrane protein Pex27 function together during peroxisome fission

AUTHORS: Lakhan Ekal and Ewald Hettema
ARTICLE TYPE: Research Article

We have now reached a decision on the above manuscript.

To see the reviewers' reports and a copy of this decision letter, please go to: https://submit-jcs.biologists.org and click on the 'Manuscripts with Decisions' queue in the Author Area. (Corresponding author only has access to reviews.)

As you will see, the reviewers raise a number of substantial criticisms that prevent me from accepting the paper at this stage. They suggest, however, that a revised version might prove acceptable, if you can address their concerns. If you think that you can deal satisfactorily with the criticisms on revision, I would be pleased to see a revised manuscript. We would then return it to the reviewers.

We are aware that you may currently be unable to access the lab to undertake experimental revisions. If it would be helpful, we encourage you to contact us to discuss your revision in greater detail. Please send us a point-by-point response indicating where you are able to address concerns raised (either experimentally or by changes to the text) and where you will not be able to do so within the normal timeframe of a revision. We will then provide further guidance. Please also note that we are happy to extend revision timeframes as necessary.

Please ensure that you clearly highlight all changes made in the revised manuscript. Please avoid using 'Tracked changes' in Word files as these are lost in PDF conversion.

I should be grateful if you would also provide a point-by-point response detailing how you have dealt with the points raised by the reviewers in the 'Response to Reviewers' box. Please attend to all of the reviewers' comments. If you do not agree with any of their criticisms or suggestions please explain clearly why this is so.

© 2023. Published by The Company of Biologists under the terms of the Creative Commons Attribution License (https://creativecommons.org/licenses/by/4.0/).

Reviewer 1

Advance summary and potential significance to field

Ekal and Hettema have demonstrated a role for the peroxin Pex27 in Vps1- (but not Dnm1-) dependent peroxisome fission in the yeast S. cerevisiae. Using single and double gene deletion mutants and mating studies, they show that Pex27 regulates peroxisome number and morphology in cooperation with the fission GTPase Vps1, but independently of the alternate fission protein Dnm1.

Overexpression of Pex27, but not Vps1, induces peroxisome proliferation in WT cells (but not pex25Δ cells) suggesting Pex27 is the limiting factor in Vps1-dependent peroxisome fission and acts downstream of Pex25. Using a catalytically-dead Vps1 mutant that accumulates at membranes, the authors show that Vps1 accumulates at puncta along peroxisome elongations in a Pex27-dependent manner, possibly via the direct interaction between Vps1 and Pex27 they have shown by pull-down assays. High-resolution imaging in cells lacking Vps1 shows Pex27 accumulation at constriction points in elongated peroxisomes supporting a model whereby Pex27 recruits Vps1 to constriction points for fission to occur. The authors also provide evidence that this model is specific to peroxisome fission for proliferation, as opposed to fission for pexophagy since pexophagy is unaffected in pex27Δ mutants, and Vps1-dependent peroxisome proliferation does not require the pexophagy receptor Atg36.

Altogether, this is a well-controlled and robust study elucidating novel molecular mechanisms underlying peroxisome fission in the yeast S. cerevisiae.
The conclusions are not over-interpreted and support the model proposed. My main concern is the data sometimes lacks quantification/statistics or is not presented clearly enough for the reader to be confident that the data shown matches the conclusions drawn.

Comments for the author

Major points The authors should provide some clarity and comparison concerning the Pex27/Pex11 proteins from different species. What are their specific functions and why are they so diverse? Since Pex27 is more specific to S. cerevisiae, the authors should discuss the broader relevance of their findings to other systems.

Several figures (e.g. S1, 2A, 5A&B) lack quantification of peroxisome numbers for all mutants, which would be useful to confirm the [lack of] differences stated between conditions as it not always obvious from the representative images. The authors should provide average peroxisome number ± SD for the mutants, to confirm the conclusions are valid. On a similar point, there are no statistical tests on any of the quantified data (Fig 1A, 2A, 2C), which would be important to add to verify that observed changes are significant. Additionally it would be useful to add quantification of peroxisome membrane structure number in vps1Δ Pex27-OE cells (Fig. 2B) to the graph in Fig. 2C – to confirm the statement that 'Pex27-OE caused an increase in peroxisomal membrane structures that was dependent upon Vps1'.

Some of the images in the figures could use additional information/different presentation to make them clearer for the reader to interpret:

- Figure 1B – what do the haploid cells look like before mating? The text states a single prelabelled peroxisome in the Pex3-containing cell. It would be useful to show an image before mating (if this is possible), since the conclusions depend on whether peroxisome fission is happening after mating.
- Figure 1D – it would be clearer if arrows were added to highlight the areas of interest in the image (e.g. Snc1 at the endosome/vacuole). It perhaps looks like the pex27Δ cells have an intermediate phenotype between the WT and vps1Δ cells in the representative images shown – is this the case, and, if so do the authors have any speculation about why?
- Figure 3A – it is not clear in the image shown if GFP-Vps1(K42A) colocalises with HcRed-PTS1 – this could be made clearer with split channels/arrows.
- Figure 4C & D – the SIM images are very impressive, but it might be useful to show HcRed-PTS1 channel on its own to more clearly demonstrate the membrane constrictions. Since Pex11 and

© 2023. Published by The Company of Biologists under the terms of the Creative Commons Attribution License (https://creativecommons.org/licenses/by/4.0/).

Pex27 are tagged with nGreen at their endogenous loci, presumably the HcRed-PTS1 signal should look the same in C & D?

The Western blot in Figure 1C lacks molecular weight markers. It is unclear where the ProtA-tagged Pex27 is coming from – is this tagged at its endogenous locus, or overexpressed? The Western blot detection is a little confusing and needs better explanation in the Methods.

Figure 3D, E needs more explanation in the text. Figure 3E is not discussed at all. The authors state in the figure legend: "Vps1-GFP preferentially precipitates Pex27-TAP over Pex22-TAP. Some background signal is observed for both Pex27-TAP and Pex22-TAP. Vps1-GFP also co-precipitates endogenous Vps1".
The IP is unclear, and as this is not a competition assay, the immunoblot saturated, showing some background, the claim "preferentially precipitates" may not be valid. Alternatively, the authors could just remove Figure 3E.

The model in Fig. 7 (and some of the data presented) implies that Pex27 is somehow involved in the constriction of elongated peroxisomes (in addition to its function as a receptor for Vps1). As the mechanism of peroxisome constriction is not well understood, a role for Pex27 in peroxisome constriction could add value to the manuscript. The authors may want to elaborate on this aspect. Are peroxisomes still able to constrict in pex27Δ/vps1Δ/dnm1Δ cells?

Based on data presented in Fig. S1, peroxisomes appear constricted in pex27Δ/dnm1Δ cells (based on mNG-PTS1 label) (?).

Do the authors suggest a role for Pex27 in the activation of the GTPase function of Vps1, as shown for human Pex11-beta? Are the functions of Pex25 and Pex27 combined in human Pex11-beta? This could be discussed with respect to the broader relevance of their findings to other systems.
Does loss of Pex27 cause loss of any other peroxisomal proteins, and does loss of Pex27 impact on Pex25?

Minor points:
- Abstract - Pex27-mNG. mNG not defined – remove?
- Axis labels missing in Fig 1A & 2A (alternatively, could be described in Figure legend).
- Typo in axis label of Fig 2C – ‘structures’ not ‘strucutres’.
- Line 143: for clarity, indicate where the data can be found
- Indicate blue staining for cell membrane in figure legends
- Figs. 1B and 6A are missing a scale bar
- Remove the black "B" in stack A, image 2 (top row) in Fig. 3B
- Do the authors have an endosome staining for Fig. 3B to confirm that the Vsp1-K42A- labelled non-peroxisomal structures are endosomes?

<u>Reviewer 2</u>

Advance summary and potential significance to field

The manuscript “The dynamin-related protein Vps1 and the peroxisomal membrane protein Pex27 function together during peroxisome fission” by Ekal and Hettema report the requirement for Pex27 in Vps1 mediated peroxisome fission. Pex27 along with Pex25 are members of the Pex11 protein family that are required for peroxisome proliferation in S. cerevisiae during growth on fatty acids. It was previously shown that Pex27 antagonist Pex25, however, in this manuscript, the authors show that Pex27 may work downstream of Pex25.
Here they propose that Pex25 is required for peroxisome elongation and Pex27 concentrates on distinct sites on the elongated peroxisomes where it recruits Vps1 to allow fission. Furthermore, they show that Pex27-Vps1 mediated peroxisome fission is independent of Vps1’s role in autophagy of peroxisomes. These findings add additional points to our current understanding of peroxisome proliferation.

© 2023. Published by The Company of Biologists under the terms of the Creative Commons Attribution License (https://creativecommons.org/licenses/by/4.0/).

Comments for the author

The manuscript will benefit by addressing the following concerns/questions:

Major points.

- An major point in this manuscript is that Pex27 interacts with Vps1 in vivo. However, the evidence provided to support this point is not convincing. The only evidence of their interaction is the immunoprecipitation experiment showing that GFP tagged Vps1 can pull down TAP-tagged Pex27. However, IP of an overexpressed protein (Vps1-GFP) cannot be used to support interaction in vivo. More supporting evidence is required to make such a statement. The authors should examine whether Pex27 can recruit the mutant Vps1K42A-GFP to the sites of fission.

- Similarly, it is not convincing that Pex27 functions downstream of Pex25. The authors suggest that Pex25 is required for peroxisome elongation. To demonstrate this point, the authors should consider overexpression Pex27 in vps1Δ/dnm1Δ/pex25Δ cells. For these experiments, a membrane marker should be used to visualize peroxisomes.

- The writing in general is esoteric as it requires a lot of knowledge of the field to understand the rationale for the experiments. This would makes it difficult for non-experts in the field, such as readers of JCS, to fully appreciate the manuscript. More description of the rationale for each experiment is needed. For example what is Pex22 and why was it used as a control?

- All the quantifications would benefit from some type of statistical analysis. Furthermore, a dot plot instead of a bar graph would give the readers a better sense of the data. This is especially so for figure 1A.

- Fig S1: the authors state that “Since only deletion of PEX27 caused a synergistic effect in dnm1Δ cells, we focussed on the role of Pex27 in peroxisome fission” (Line 152-153). However, in the representative images shown, dnm1Δ/pex25Δ and dnm1Δ/pex34Δ also show a further reduction in peroxisomes compared to dnm1Δ cells and also for the single deletion pex25Δ and pex34Δ cells. Quantification of these cells would be beneficial.

- Fig 1B: to exclude the possible role of Pex25 in Vps1 dependent fission, a cross between vps1Δ/dnm1Δ/pex25Δ +mNG-PTS1 and pex3Δ+mKate2-PTS1 should be done.

- Line 186-187: the authors claimed that “Pex27 is specifically required for Vps1-dependent peroxisome fission”. However, the same group has previously shown that DNM1 can substitute for Vps1 in peroxisome fission (Motley 2007). If this is the case, then does the substitution of DNM1 also require Pex27?

-Fig 2A: to further show that Pex27 is required for Vps1-dependent peroxisome fission, the authors need to examine whether VPS1 overexpression can restore peroxisomes in vps1Δ/pex27Δ cells. Control of this will be overexpression of DNM1, Pex25, and EP in vps1Δ/pex27Δ cells.

- Fig 2B: If Pex27 is required for Vps1 recruitment for peroxisome fission, why are the Pex11-mNG labeled peroxisomes larger in WT PEX27OE compared to vps1Δ + PEX27OE? Shouldn’t the vps1Δ + PEX27OE cells have larger peroxisomes given that there is no fission?

- Line 215-216: if the activity of Pex27 depends upon Pex25, is Pex25 also required for Vps1 dependent peroxisome fission? If this is the case, why is Pex27 the limiting factor, but not Pex25?

- Quantification of peroxisome adundance in Figures 5A and B would be helpful to interpret the data.

Minor points

- Fig 1A The X-axis label is needed either in the figure or legend.
- Line 252: check “HcRed-0PTS1”

© 2023. Published by The Company of Biologists under the terms of the Creative Commons Attribution License (https://creativecommons.org/licenses/by/4.0/).

- In Figure 1B is it possible that the peroxisomes are elongated but constricted peroxisomes in panel I and II?
Using a membrane marker may help in resolving peroxisomal membrane structure.

Reviewer 3

Advance summary and potential significance to field

The manuscript by Ekal and Hettema illustrates potential molecular detail for the process of peroxisomal fission in budding yeasts. The study reveals that in order to maintain constitutive peroxisomal division activity in the cells undergoing exponential growth, a specific dynamin-related protein (i.e., Vps1p) works in play together with selective peroxisomal membrane components (e.g., Pex27p). Pex27p, which recruits Vps1p onto the peroxisome surface, remodels the organellar membranes and helps facilitate membrane scission. Interestingly, Pex27p does not seem to be involved in either pexophagy-related or Dnm1p-dependent peroxisomal division processes. Thus, the authors suggest that Pex27p functions as a unique rate liming factor in controlling peroxisomal division and maintenance, especially in mitotically-active cells.

The manuscript is well-written and provides key molecular mechanism for peroxisomal biogenesis and homeostasis, thereby adding new insights into the field of organellar biology. However, to this reviewer the current study lacks some of essential data analyses with imaging quantification. Thus, to further strengthen the study's new findings and to fulfill the journal's publication standard, the authors should address several concerns listed below.

Comments for the author

Major concerns:

Figure 1B's mating assay demonstrates Vps1p's selective involvement in peroxisomal division and is supported by Figure 1A and S1. However, without having proper quantification of image data in Figure 1B the authors' claim for the distinct interaction between Vps1p and Pex27p is somewhat subjective and less convincing. Some of GFP signals are too saturated and the projected z-stack images make the readers difficult to identify discrete peroxisome structures. Therefore, as shown in Figure 1A, it is highly recommended to present additional imaging analysis data (e.g., changes in peroxisome number and length). Also, the additional control data from MatA pex3delta cells with no DNM1 is essential to be included in the study in order to rule out potential interplay between Pex27p and Dnm1p (or Fis1p) in the process of peroxisomal division.

With similar reason in the above, Figure 2 also needs proper quantification. Indeed, the authors themselves referred quantitative values (i.e., peroxisome number) to describe cellular changes in peroxisomal fission activity upon indicated gene manipulations in the result section ("although overexpression of Vps1 restores peroxisome number in vps1.."). Data shown by only representative images with poor resolution in space and time is insufficient to be conclusive. Thus, the authors need to provide properly quantified data to help the readers' interpretation.

In addition, Figure 2A is missing the representative images from WT cells harboring mNG-PTS1 with and without VPS1 (or DNM1) overexpression. Pex11-mNG images from WT cells with and without VPS1 overexpression in Figure 2B is not appropriate to make the authors' point in that Vps1p, but not Dnm1p, is a rate limiting factor for peroxisomal fission. This is because the authors pointed out potential localization differences between peroxisomal matrix marker (i.e., mNG-PTS1) versus the membrane proteins (e.g., Pex11-mNG) in the text. Thus, for an appropriate comparison, WT cells harboring either Pex11-mNG or mNG-PTS with overexpressed Vps1p/Dnm1p should be included in Figure 2A with proper quantification.

Furthermore, Figure 2C should also include the quantification data from vps1 delta cells harboring Pex11-mNG with PEX27 overexpression to support Figure 2B and to clearly demonstrate Vps1p's requirement for Pex27p-mediated increase in peroxisomal abundance.

© 2023. Published by The Company of Biologists under the terms of the Creative Commons Attribution License (https://creativecommons.org/licenses/by/4.0/).

Figure 3A also needs proper quantification for the changes in peroxisome number to support that Vps1-GFP, but not Vps1-K42A-GFP induces peroxisomal fission in cells lacking either VPS1 only or both VPS1 and DNM1.

First revision

Author response to reviewers' comments

The authors would like to thank the referees for their efforts and suggestions and we have taken them into serious consideration, with many implemented into the revised manuscript. As you will see from the manuscript, we have added more information in the introduction and rewritten the beginning of the result section and discussion.
Please find below my comments on each point raised by the referees. In the manuscript, I have indicated the major textual changes in blue.

Reviewer 1 Comments for the Author:

Major points

Comment:The authors should provide some clarity and comparison concerning the Pex27/Pex11 proteins from different species. What are their specific functions and why are they so diverse?

Answer: In the introduction we have now included additional information with respect to the Pex11 protein family:

page 4-5: This family of proteins is conserved among eukaryotes and has been linked to peroxisome division (Honsho et al., 2016; Schrader et al., 2016) and some family members have been implicated in *de novo* formation of peroxisomes (Huber et al., 2012, Chang et al., 2015). ……………Pex25 is a fungal innovation (Chang et al., 2015). Its paralogue Pex27 ….

Comment: Since Pex27 is more specific to S. cerevisiae, the authors should discuss the broader relevance of their findings to other systems.

Answer: The broader relevance of this work is that the different peroxisome fission processes each rely on different accessory proteins. This is addressed in the discussion. In addition, it is of interest for readers outside the peroxisome field to see that common membrane remodelling factors such as Vps1 can act on different membranes under different conditions through a variety of factors. We also now discuss our findings in the context of Pex11beta.

page 13-15: In mammals and the yeast *H. polymorpha*, the tubulation of the peroxisomal membrane is induced by Pex11β/HpPex11.………We cannot exclude additional later roles for Pex27 in Vps1-dependent fission, for instance in the regulation of Vps1 GTPase activity analogous to *H. polymorpha* Pex11 and human Pex11β in the regulation of Dnm1/Drp1 (Williams et al., 2015).

Comment: Several figures (e.g. S1, 2A, 5A&B) lack quantification of peroxisome numbers for all mutants, which would be useful to confirm the [lack of] differences stated between conditions as it not always obvious from the representative images. The authors should provide average peroxisome number ± SD for the mutants, to confirm the conclusions are valid. On a similar point, there are no statistical tests on any of the quantified data (Fig 1A, 2A, 2C), which would be important to add to verify that observed changes are significant. Additionally, it would be useful to add quantification of peroxisome membrane structure number in vps1Δ Pex27-OE cells (Fig. 2B) to the graph in Fig. 2C - to confirm the statement that ‘Pex27-OE caused an increase in peroxisomal membrane structures that was dependent upon Vps1’.

Answer: We have now included more quantitative data throughout the manuscript and included statistical analysis where required.

© 2023. Published by The Company of Biologists under the terms of the Creative Commons Attribution License (https://creativecommons.org/licenses/by/4.0/).

Comment: Some of the images in the figures could use additional information/different presentation to make them clearer for the reader to interpret:
•Figure 1B – what do the haploid cells look like before mating? The text states a single prelabelled peroxisome in the Pex3-containing cell. It would be useful to show an image before mating (if this is possible), since the conclusions depend on whether peroxisome fission is happening after mating.

Answer: An extra panel (panel IV) has now been included in Figure 1B, arrows indicate haploid cells.

Comment: Figure 1D – it would be clearer if arrows were added to highlight the areas of interest in the image (e.g. Snc1 at the endosome/vacuole). It perhaps looks like the pex27Δ cells have an intermediate phenotype between the WT and vps1Δ cells in the representative images shown – is this the case, and, if so, do the authors have any speculation about why?

Answer: We have now included white arrows in the figure to indicate areas of polarised growth, (Buds and bud neck) where we see increased GFP signal. In wt and pex27 cells the label inside cells is weak, including vacuolar labelling indicated by red arrow, compared to the signal at the plasma membrane in the bud and bud neck (in cells around the time of cytokinesis). In vps1 cells no clear polarised plasma membrane signal is observed and most GFP is found intracellularly. We have included a sentence in the text: As secretion is a polarised process in *S. cerevisiae*, GFP-Snc1 strongly labels the plasma membrane in buds and in the bud neck in cells prior to cytokinesis (Lewis et al., 2000).

Comment: Figure 3A – it is not clear in the image shown if GFP-Vps1(K42A) colocalises with HcRed-PTS1 – this could be made clearer with split channels/arrows.

Answer: The main purpose of Fig 3A is to show that Vps1K42A-GFP does not restore peroxisome fission in vps1Δdnm1Δ cells. We have now included quantitative data to support this conclusion. The weak signals for Vps1K42A- GFP colocalising with peroxisomes are frequently difficult to see in merges of Z stacks as shown in Fig. 3a. We have split the channels, red/green and some colocalisation may be present. But since it is a merge of different focal planes, apparent colocalisation could be caused by signal overlap in different planes of the stack. This is further analysed in fig. 3b where individual focal planes are analysed and where we show proper colocalisation within single focal planes.

Comment: Figure 4C & D – the SIM images are very impressive, but it might be useful to show HcRed-PTS1 channel on its own to more clearly demonstrate the membrane constrictions.

Answer: We have now done this.

Comment: Since Pex11 and Pex27 are tagged with nGreen at their endogenous loci, presumably the HcRed-PTS1 signal should look the same in C & D?

Answer: Indeed, they do look punctate but with Pex11-mNG labelling, we did observe the occasional peroxisome extension without any matrix protein labelling.

Comment: The Western blot in Figure 1C lacks molecular weight markers. It is unclear where the ProtA-tagged Pex27 is coming from – is this tagged at its endogenous locus, or overexpressed?The Western blot detection is a little confusing and needs better explanation in the Methods.

Answer: Molecular weights have now been added. ProtA tagged Pex27 was expressed under control of its own promoter on a yeast centromeric plasmid (ARS1/CEN4) expressed in WT, vps1Δ and pex3Δ cells. These ARS1/CEN4 plasmids are present at 1-2 copies per cell (see for instance Falcon and Aris, 2000). We also explain the Western blot detection in more detail in Materials and Methods.

Comment: Figure 3D, E needs more explanation in the text. Figure 3E is not discussed at all. The authors state in the figure legend: "Vps1-GFP preferentially precipitates Pex27-TAP over Pex22-TAP. Some background signal is observed for both Pex27-TAP and Pex22-TAP. Vps1-GFP also co-precipitates endogenous Vps1".

© 2023. Published by The Company of Biologists under the terms of the Creative Commons Attribution License (https://creativecommons.org/licenses/by/4.0/).

The IP is unclear, and as this is not a competition assay, the immunoblot saturated, showing some background, the claim "preferentially precipitates" may not be valid. Alternatively, the authors could just remove Figure 3E.
Answer: We have removed Fig 3E and have now described the strains and western blot detection for this experiment in more detail in the section of coimmunoprecipitation.

A Pex27-TAP tagged strain (Ghaemmaghami, S. et al. 2003) was transformed with a centromeric plasmid encoding Vps1-GFP under control of its endogenous promoter and as negative control, a plasmid encoding GFP-PTS1 under control of the strong constitutive *TPI1* promoter. Using GFP-TRAP, GFP-PTS1 and Vps1-GFP were precipitated. Pex27-TAP and endogenous Vps1 co-precipitated with Vps1-GFP but not with GFP-PTS1. This indicates that Pex27 and Vps1 can physically interact *in vivo* and that Vps1-GFP assembles into Vps1 oligomers (Fig. 3D).

Comment: The model in Fig. 7 (and some of the data presented) implies that Pex27 is somehow involved in the constriction of elongated peroxisomes (in addition to its function as a receptor for Vps1). As the mechanism of peroxisome constriction is not well understood, a role for Pex27 in peroxisome constriction could add value to the manuscript. The authors may want to elaborate on this aspect. Are peroxisomes still able to constrict in pex27Δ/vps1Δ/dnm1Δ cells?

Answer: Sorry about the confusion and we have now amended the model. We now show that constricted areas are still present in dnm1/pex27 and vps1/dnm1/pex27 cells (new panels, Fig. 4G,H). This means that initial constriction does not require Pex27. This is also in line with our observations that Dnm1 dependent fission can still proceed in the absence of Pex27 (Fig. 2A). Overexpressed Pex27 and Pex27-GFP appear to extend the constrictions between bulbous parts of peroxisomes (see figure 3E and 3F, respectively).
This strongly supports our SIM data that Pex27-mNG accumulates in between the bulbous parts of peroxisomes, i.e. on tubular, constricted membranes. We therefore propose that Pex27 accumulates at constricted areas but is not required for the initial constriction. Our working model in figure 7 now states:
The multistep process of peroxisome fission is initiated by (1) Pex25-dependent protrusion and elongation of the peroxisomal membrane. (2) Import of proteins allows peroxisomes to grow and Pex27 concentrates on the highly curved membrane at tubular, constricted regions between bulbous areas. Vps1 associates with peroxisomes dependent on Pex27....

Comment: Do the authors suggest a role for Pex27 in the activation of the GTPase function of Vps1, as shown for human Pex11-beta? Are the functions of Pex25 and Pex27 combined in human Pex11-beta? This could be discussed with respect to the broader relevance of their findings to other systems.

This is an interesting point but we really can not suggest this. We have not explored whether Pex27 can function as a GAP for Vps1 as has been shown in vitro for Pex11 in the regulation of the GTPase activity of Dnm1/Drp1. If the sole function of Pex27 was to induce the GTPase activity of Vps1, we would expect to see Vps1 still being associated with peroxisomes in pex27 deficient cells. However, we observed a dependency of Vps1-K42A to associate with peroxisomes on Pex27, suggesting that Pex27 acts before GTP hydrolysis.
Whether Pex27 has multiple functions as has been reported for Pex11 is possible but unexplored. See new part of discussion above.

Does loss of Pex27 cause loss of any other peroxisomal proteins, and does loss of Pex27 impact on Pex25?

We have not tested whether peroxisomal proteins are deficient in pex27 cells but pex27 cells grow normally on oleate media (Tam et al., 2003; Rottensteiner et al., 2003;Huber et al., 2012) suggesting that there are no major biogenesis or import defects. Pex25 is stable in pex27 cells (Fig S3G).Huber et al (2012) showed that Pex11 and thiolase levels are not affected in pex27 cells. So overall, there are no indications of general effects on peroxisomal protein levels.

Minor points:
- Abstract - Pex27-mNG. mNG not defined – remove? **Removed**
- Axis labels missing in Fig 1A & 2A (alternatively, could be described in Figure legend). **Added to**

© 2023. Published by The Company of Biologists under the terms of the Creative Commons Attribution License (https://creativecommons.org/licenses/by/4.0/).

figure
• Typo in axis label of Fig 2C – 'structures' not 'strucutres'. **Changed to Pex11-mNG puncta**
• Line 143: for clarity, indicate where the data can be found. **We changed the text in the result section by leaving it out there but then we included the following in the Materials and Methods, page 19.**

Certain phenotypes are sensitive to cell growth rate. For instance, peroxisome inheritance defects are compensated for by de novo formation and the number of cells without peroxisomes increases in exponential growing cultures vs stationary phase cultures (Motley, Ward and Hettema. 2009). Likewise, peroxisome number in pexophagy mutants is affected by growth rate (Nuttall et al., 2014, 2015). Therefore, in subsequent experiments,.......

• Indicate blue staining for cell membrane in figure legends. **Done**
• Figs. 1B and 6A are missing a scale bar **Done**
• Remove the black "B" in stack A, image 2 (top row) in Fig. 3B **Done**

Comment:Do the authors have an endosome staining for Fig. 3B to confirm that the Vsp1-K42A-labelled non-peroxisomal structures are endosomes?

Answer: No but we have used a pulse chase with FM4-64. This is a lipophilic dye that is endocytosed and transported via endosomes to the vacuole. In vps1 deficient cells this process is delayed and vacuoles are fragmented. Nonetheless we did see colocalisation with the bright Vps1K42A-GFP puncta and FM4-64 after 5-10 min. We have not included the data into to this manuscript but included it here in the referees report. For more details of localisation of Vps1 K42A-GFP on endosomes we refer to Tornabene et al., 2020 and Varlakhanova et al., 2018.

© 2023. Published by The Company of Biologists under the terms of the Creative Commons Attribution License (https://creativecommons.org/licenses/by/4.0/).

Vps1 K42A-GFP in *vps1/dnm1* cells

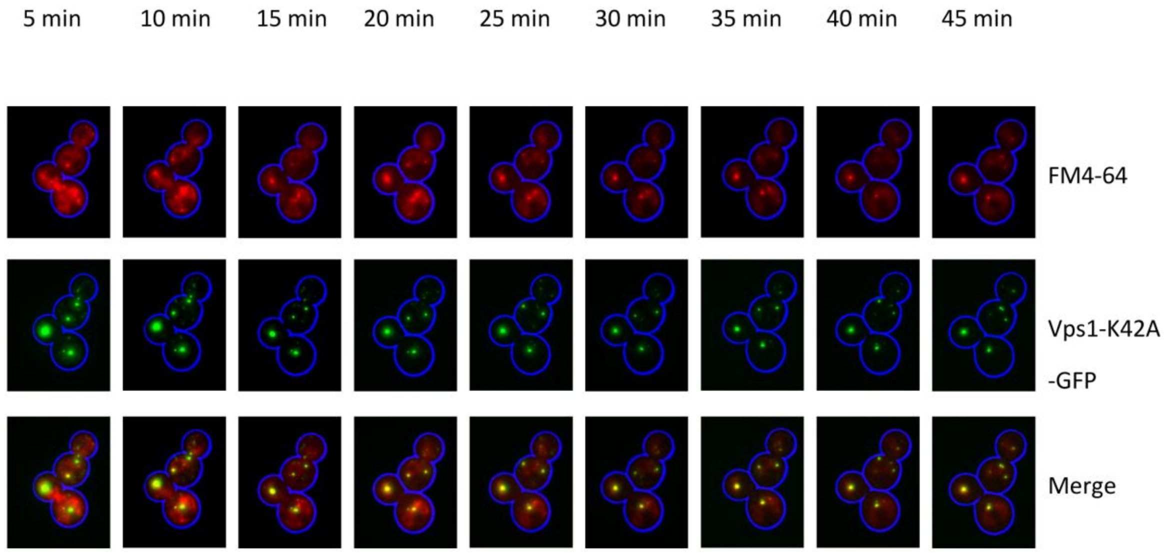

Negative control for the bleed through of Vps1 K42A-GFP in cells not labelled with FM4-64

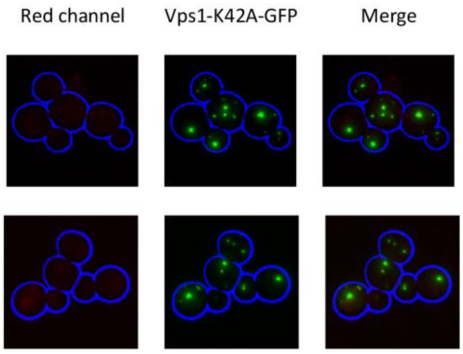

Reviewer 2 Comments for the Author:

The manuscript will benefit by addressing the following concerns/questions:

Major points:

Comments: A major point in this manuscript is that Pex27 interacts with Vps1 in vivo. However, the evidence provided to support this point is not convincing. The only evidence of their interaction is the immunoprecipitation experiment showing that GFP-tagged Vps1 can pull down TAP-tagged Pex27. However, IP of an overexpressed protein (Vps1-GFP) cannot be used to support interaction in vivo. More supporting evidence is required to make such a statement.

Answer: We are sorry about the confusion of the experimental set up. We have now described the Co-IP experiment in more detail and made clear that Pex27- TAP was expressed from its endogenous genomic locus and that Vps1-GFP was expressed from a CEN4/ARS1 plasmid under control of its own promoter (plasmid pKA1078). These plasmids are present at 1-2 copies per cell. This would represent one extra copy of the Vps1 gene compared to in WT cells.
With our experiment we show that under our conditions a small fraction of Pex27 interacts with a small fraction of Vps1.

Comment: The authors should examine whether Pex27 can recruit the mutant Vps1K42A-GFP to the sites of fission.

© 2023. Published by The Company of Biologists under the terms of the Creative Commons Attribution License (https://creativecommons.org/licenses/by/4.0/).

Answer: We have tried using SIM but unfortunately, Vps1 K42A-GFP signals on peroxisomes are too weak for SIM. We also tried BiFC between Pex27 and Vps1. However, Pex27 signals were only clear upon prolonged overexpression. However, we clearly observe Vps1 K42A-GFP signal at constricted areas of the peroxisomal membrane, juxtaposed to bulbous parts in vps1/dnm1 cells (Fig 3B, now indicated with a white arrow). Recruitment to peroxisomes is dependent on Pex27 (Fig3c)

Comment: Similarly, it is not convincing that Pex27 functions downstream of Pex25. The authors suggest that Pex25 is required for peroxisome elongation. To demonstrate this point, the authors should consider overexpression Pex27 in vps1Δ/dnm1Δ/pex25Δ cells. For these experiments, a membrane marker should be used to visualise peroxisomes.

Answer: We did the experiment proposed by the referee (fig. S3H,I). Overexpression of Pex27 in vps1/dnm1/pex25 cells does not restore the formation of the elongated tubules normally found in vps1/dnm1 cells.

Comments: The writing in general is esoteric as it requires a lot of knowledge of the field to understand the rationale for the experiments. This would make it difficult for non-experts in the field, such as readers of JCS, to fully appreciate the manuscript. More description of the rationale for each experiment is needed. For example, what is Pex22 and why was it used as a control?

Answer: Upon suggestion of reviewer 1 we have now removed Pex22. We also have rewritten parts of the manuscript to include more introduction and context.

Comments: All the quantifications would benefit from some type of statistical analysis. Furthermore, a dot plot instead of a bar graph would give the readers a better sense of the data. This is especially so for figure 1A.

Answer: We have now included more quantitative data, including dot plots throughout the manuscript and included statistical analysis.

Comment:Fig S1: the authors state that “Since only deletion of PEX27 caused a synergistic effect in dnm1Δ cells, we focussed on the role of Pex27 in peroxisome fission” (Line 152-153). However, in the representative images shown, dnm1Δ/pex25Δ and dnm1Δ/pex34Δ also show a further reduction in peroxisomes compared to dnm1Δ cells and also for the single deletion pex25Δ and pex34Δ cells. Quantification of these cells would be beneficial.

Answer: Quantitation is now included. We meant to indicate that to identify factors specifically required for Vps1-dependent fission, the absence of this factor in a *dnm1* mutant should result in a strong reduction in peroxisome number. A double mutant of this factor with *vps1* should not be more severe than the *vps1* single mutant. We have now rewritten the first section of the results to explain this more clearly. Page 7:

In most organisms studied, peroxisome fission relies on a single DRP, Dnm1/Drp1. Fungal Dnm1/Drp1 acts in concert with Fis1, Mdv1 and as shown in *H. polymorpha*, also Pex11. Peroxisome fission in *S. cerevisiae*, mainly relies on the Drp, Vps1, and to a lesser extent, Dnm1. As Vps1 dependent fission is not dependent upon Fis1, Mdv1, and its paralogue Caf4 (Nagotu et al., 2008; Motley et al., 2008) we set out to identify factors specifically required for Vps1-dependent peroxisome fission. We generated double gene deletion mutants of *PEX11*, *PEX25*, *PEX27* and *PEX34* with either a *VPS1* or a *DNM1* deletion and compared peroxisome number in each of the strains. A factor specifically required for Vps1-dependent fission is expected to 1) show a strong decrease in peroxisome number when deleted on its own, as is observed in *vps1⊗* cells, 2) have no further decrease in peroxisome number upon *VPS1* deletion and 3) show a decrease in peroxisome number upon *DNM1* deletion.

Comment: Fig 1B: to exclude the possible role of Pex25 in Vps1 dependent fission, a cross between vps1Δ/dnm1Δ/pex25Δ +mNG-PTS1 and pex3Δ+mKate2-PTS1 should be done.

© 2023. Published by The Company of Biologists under the terms of the Creative Commons Attribution License (https://creativecommons.org/licenses/by/4.0/).

Answer: We do not exclude a role for Pex25 in Vps1 dependent fission as, on the contrary, a pex25 deletion further affects peroxisome number in vps1 cells. Also overexpression of Vps1 in pex25 cells does not increase peroxisome number (See new Fig. S2B).

Comment: Line 186-187: the authors claimed that “Pex27 is specifically required for Vps1-dependent peroxisome fission”. However, the same group has previously shown that DNM1 can substitute for Vps1 in peroxisome fission (Motley 2007). If this is the case, then does the substitution of DNM1 also require Pex27?

Answer: Overexpression of Dnm1 in pex27 and dnm1/pex27 cells increases peroxisome number, implying that Dnm1 can divide peroxisomes independent of Pex27. We also explain more in the introduction that Dnm1 dependent peroxisome fission relies on Fis1, Mdv1, Caf4 (see also Motley et al., 2008) and in the yeast Hansenula polymorpha on Pex11.

-

Comment:Fig 2A: to further show that Pex27 is required for Vps1-dependent peroxisome fission, the authors need to examine whether VPS1 overexpression can restore peroxisomes in vps1Δ/pex27Δ cells. Control of this will be overexpression of DNM1, Pex25, and EP in vps1Δ/pex27Δ cells.

Answer: We consider the Vps1 overexpression in pex27 cells similar to the overexpression of Vps1 in vps1/pex27 cells. If the reviewer is worried about lack of activity of the Vps1 overexpression construct we refer to the complementation by this construct in vps1/dnm1 cells (Fig. 2a).

Comment:Fig 2B: If Pex27 is required for Vps1 recruitment for peroxisome fission, why are the Pex11-mNG labeled peroxisomes larger in WT PEX27OE compared to vps1Δ + PEX27OE? Shouldn’t the vps1Δ + PEX27OE cells have larger peroxisomes given that there is no fission?

Answer: The reviewer is correct that we expect smaller peroxisomes in WT +Pex27OE than in vps1+ Pex27OE. In some of the WT (Fig 1A, Fig S1) and in WT +pex27OE cells we see some clustering or larger peroxisomal structures. Unique to WT+PEX27OE however, is the presence of a large number (>10/cell) of very small/faint fluorescent structures which are absent from vps1+ Pex27OE cells. Also, the tubulation observed in vps1 cells is more difficult to visualise as the tubules extending from the peroxisomes are rather faintly labelled with Pex11-mNG as also described for vps1/dnm1 cells + PEX27OE in Figure 4E. We have now included a different panel of vps1 + PEX27 OE cells and we have added an inset that we overexposed to show the extension from the peroxisome.

Comment:Line 215-216: if the activity of Pex27 depends upon Pex25, is Pex25 also required for Vps1 dependent peroxisome fission? If this is the case, why is Pex27 the limiting factor, but not Pex25?

With a limiting factor we mean that the level of Pex27 is limiting for Vps1 dependent fission. Higher levels of Pex27 result in increased Vps1 dependent peroxisome fission. Pex25 overexpression does not result in an increased number of peroxisomes but in elongated peroxisomes (see for instance Huber et al., 2012) and our own observations not included in this manuscript.

- Quantification of peroxisome abundance in Figures 5A and B would be helpful to interpret the data.
We agree, and this has been done now.

Minor points

- Fig 1A The X-axis label is needed either in the figure or legend. **Done**

- Line 252: check “HcRed-0PTS1” **Now changed**

- In Figure 1B is it possible that the peroxisomes are elongated but constricted peroxisomes in panel I and II? Using a membrane marker may help in resolving peroxisomal membrane structure.

© 2023. Published by The Company of Biologists under the terms of the Creative Commons Attribution License (https://creativecommons.org/licenses/by/4.0/).

Answer: Normally, we can clearly visualise the tubular extension within the non divided peroxisome with epifluorescence microscopy. In addition the puncta do not disperse as observed in panel II and III. This is clear from panel IV, V and VI, especially when using the very bright and photostable marker mNG-PTS1. However, we can not completely rule out that Vps1 has constricted potential connections so much in panel I and II so that we can not see them any longer. We have now used the term 'membrane remodelling' instead of fission to indicate the morphology change we see early upon mating.

Reviewer 3 Comments for the Author:
Major concerns:
Comment:Figure 1B's mating assay demonstrates Vps1p's selective involvement in peroxisomal division and is supported by Figure 1A and S1. However, without having proper quantification of image data in Figure 1B, the authors' claim for the distinct interaction between Vps1p and Pex27p is somewhat subjective and less convincing. Some of GFP signals are too saturated and the projected z-stack images make the readers difficult to identify discrete peroxisome structures. Therefore, as shown in Figure 1A, it is highly recommended to present additional imaging analysis data (e.g., changes in peroxisome number and length).

Answer:We have now included numbers in the text and decreased the level of green signal in some of the panels.

Comment:Also, the additional control data from MatA pex3delta cells with no DNM1 is essential to be included in the study in order to rule out potential interplay between Pex27p and Dnm1p (or Fis1p) in the process of peroxisomal division.

Answer:Dnm1 does not contribute to fission in this assay as can be seen from mating vps1/dnm1 cells with vps1/pex3 cells. Upon this mating, no fission of peroxisomes is observed in large zygotes (panel V and in Motley and Hettema, Fig.6 (2007). We needed to use overexpression of Dnm1 to induce fission in this background, (Fig 6c Motley and Hettema, 2007) and see also Motley et al., 2008). Since there is no statistically significant difference between peroxisome number in pex27, vps1 and pex27/vps1 cells (Fig. 1A), Dnm1 seems to acts independent of Pex27. We refer to Fig. 2A where we show that overexpression of Dnm1 rescues the *PEX27* deletion, indicating that Dnm1 can indeed act independent of Pex27.

Comment:With similar reason in the above, Figure 2 also needs proper quantification. Indeed, the authors themselves referred quantitative values (i.e., peroxisome number) to describe cellular changes in peroxisomal fission activity upon indicated gene manipulations in the result section ("although overexpression of Vps1 restores peroxisome number in vps1.."). Data shown by only representative images with poor resolution in space and time is insufficient to be conclusive. Thus, the authors need to provide properly quantified data to help the readers' interpretation.

Answer:This has now been done and included in the figures.

Comment: Figure 2A is missing the representative images from WT cells harboring mNG-PTS1 with and without VPS1 (or DNM1) overexpression. Pex11-mNG images from WT cells with and without VPS1 overexpression in Figure 2B is not appropriate to make the authors' point in that Vps1p, but not Dnm1p, is a rate limiting factor for peroxisomal fission.

This is because the authors pointed out potential localization differences between peroxisomal matrix marker (i.e., mNG-PTS1) versus the membrane proteins (e.g., Pex11-mNG) in the text. Thus, for an appropriate comparison, WT cells harboring either Pex11-mNG or mNG-PTS1 with overexpressed Vps1p/Dnm1p should be included in Figure 2A with proper quantification.

Answer:We have now included the effect of Vps1 and Dnm1 overexpression in WT cells using a matrix protein marker. See Fig. S2A.

Comment:Figure 2C should also include the quantification data from vps1 delta cells harboring Pex11-mNG with PEX27 overexpression to support Figure 2B and to clearly demonstrate Vps1p's requirement for Pex27p-mediated increase in peroxisomal abundance.

© 2023. Published by The Company of Biologists under the terms of the Creative Commons Attribution License (https://creativecommons.org/licenses/by/4.0/).

Answer:We have now included the quantitative data.

Comment:Figure 3A also needs proper quantification for the changes in peroxisome number to support that Vps1-GFP, but not Vps1-K42A-GFP induces peroxisomal fission in cells lacking either VPS1 only or both VPS1 and DNM1.

Answer:We have now provided the quantification in vps1/dnm1 cells transformed with empty plasmids (EP), GFP tagged Vps1 WT and Vps1-K42A behind the Vps1 promoter in (Fig. 3A).

Second decision letter

MS ID#: JOCES/2020/246348

MS TITLE: The dynamin-related protein Vps1 and the peroxisomal membrane protein Pex27 function together during peroxisome fission

AUTHORS: Lakhan Ekal, Abdulaziz M.S Alqahtani, and Ewald Hettema
ARTICLE TYPE: Research Article

I am happy to tell you that your manuscript has been accepted for publication in Journal of Cell Science, pending standard ethics checks.

Reviewer 1

Advance summary and potential significance to field

This is a well-controlled and robust study elucidating novel molecular mechanisms underlying peroxisome fission in the yeast S. cerevisiae.

My main concerns related to quantification of data/statistics and clarity of data presentation have been fully addressed in the revised version.

Comments for the author

I have no further comments and would like to thank the authors for their thorough revisions.

Reviewer 2

Advance summary and potential significance to field

See below

Comments for the author

In this revised version authors have addressed all my concerns.

© 2023. Published by The Company of Biologists under the terms of the Creative Commons Attribution License (https://creativecommons.org/licenses/by/4.0/).
